# Development and In Vitro Evaluation of Atorvastatin and Rutin Co-Loaded Nanoliposomes for Enhanced Anti-Inflammatory and Cytotoxic Efficacy

**DOI:** 10.3390/ijms27188216

**Published:** 2026-09-15

**Authors:** Ali Al-Samydai, Violet Kasabri, Hanan Azzam, Maha N. Abu Hajleh, Said Moshawih, Hamdi Al Nsairat, Lidia Al-Halaseh, Heba Banat, Zahraa Al-Zubaidy, Zain Al-Tarawneh, Yusuf Al-Hiari, Thaqif El Khassawna, Rana Elstaty, Dina Abu AlSaman, Emad A. S. Al-Dujaili

**Affiliations:** 1 Department of Pharmaceutics and Pharmaceutical Technology, Pharmacological and Diagnostic Research Centre, Faculty of Pharmacy, Al-Ahliyya Amman University, Amman 19328, Jordan; h.azzam@ammanu.edu.jo (H.A.); zahraa94f@gmail.com (Z.A.-Z.); zainaltarawneh98@gmail.com (Z.A.-T.);; 2 School of Pharmacy, The University of Jordan, Amman 11942, Jordan; violetk70@gmail.com (V.K.); hiary@ju.edu.jo (Y.A.-H.); 3 Department of Cosmetic Science, Pharmacological and Diagnostic Research Centre, Faculty of Allied Medical Sciences, Al-Ahliyya Amman University, Amman 19328, Jordan; m.abuhajleh@ammanu.edu.jo; 4 Department of Pharmaceutical Chemistry, Faculty of Pharmacy, Mutah University, Al-Karak 61710, Jordan; drhalaseh@mutah.edu.jo; 5 Faculty of Medicine, Justus Liebig University, Giessen 35390, Germany; thaqif.el.khassawna@ges.thm.de; 6 Institute of Pancreatic Disease, Department of Gastroenterology, Semmelweis University, Üllőiút 26, Budapest 1085, Hungary; 7 Centre for Cardiovascular Science, Queen’s Medical Research Institute, University of Edinburgh, Edinburgh EH8 9YL, UK

**Keywords:** nanoliposomes, atorvastatin, rutin, drug delivery, antioxidant activity, anti-inflammatory, selective cytotoxicity, drug repurposing, cancer therapy

## Abstract

Liposomal drug-delivery systems can improve the formulation performance of poorly soluble compounds by enhancing aqueous dispersion, protecting encapsulated agents, and modifying release behavior. Co-encapsulation of pharmacologically distinct compounds may provide a formulation strategy for comparing combined delivery with a single agent nanoliposomal system. This study aimed to develop and characterize atorvastatin–rutin co-loaded nanoliposomes and to compare their antioxidant, anti-inflammatory, and SRB-based cytotoxic activity with the corresponding free-drug and single-loaded nanoliposomal formulations. Nanoliposomes were prepared by thin-film hydration and characterized by particle size, polydispersity index, zeta potential, encapsulation efficiency, lyophilization-associated retention of encapsulation efficiency, morphology, and in vitro release. A reverse-phase HPLC method was validated for simultaneous atorvastatin and rutin quantification, and lyophilized formulations were evaluated for retention of encapsulation efficiency. In vitro assays included DPPH radical scavenging, nitrite inhibition in LPS-stimulated RAW 264.7 macrophages, and SRB-based cytotoxicity screening across human cancer cell lines and normal periodontal ligament fibroblasts. The co-loaded nanoliposomes achieved encapsulation efficiencies of 88.46% for atorvastatin and 81.74% for rutin; after lyophilization, encapsulation efficiency decreased to 72.31% for atorvastatin and 76.63% for rutin. The nanoliposomal formulations showed measurable DPPH radical-scavenging activity, nitrite-inhibition activity in LPS-stimulated macrophages, and SRB-based antiproliferative activity in several cancer cell lines, while showing no detectable cytotoxicity toward PDL fibroblasts within the tested concentration range. With exquisite similarity to apoptogenic Anti-VEGF antiangiogenesis chemotherapeutic efficacies ofcisplatin; nanoliposomal atorvastatin and co-loaded atorvastatin with rutin were remarkably comparable (in descending order of human VEGF mitigations) in mammary T47D> uterine cervix HeLa> lung A549 adherent monolayers post 72 h incubations. These findings support further investigation of atorvastatin–rutin co-loaded nanoliposomes as an in vitro formulation platform; however, formal synergy analysis, cellular uptake studies, mechanistic assays, pharmacokinetic evaluation, and in vivo safety testing remain necessary. Further in vivo studies are required to determine pharmacokinetic behavior, tissue distribution, therapeutic relevance, and systemic safety.

## 1. Introduction

Cancer remains a major global problem. In 2020, there were nearly 10 million deaths and 19.3 million new cases [1]. Even with better early detection and treatment, the number of cases continues to grow [2,3]. Chemotherapy works effectively, but it lacks specificity and also harms healthy tissue, leading to serious side effects [3]. In addition, drug resistance often limits long-term success [4]. This shows the urgent need for safer and more targeted cancer treatments.

Drug repurposing is becoming popular. It involves finding new cancer uses for existing medications [4,5]. This approach saves time and costs by skipping much of the early safety and pharmacokinetic testing [6,7]. For example, atorvastatin, a common cholesterol drug, has been shown in several studies to fight cancer cells, slow their growth, and stop new blood vessels from forming [8,9,10,11,12,13,14,15,16,17,18,19,20,21,22,23]. Yet, using it for cancer is not straightforward. It requires higher doses than cholesterol and does not dissolve well in water. Moreover, it is quickly metabolized by the liver, which can increase the risk of side effects [24,25].

Rutin, a natural flavonoid, has antioxidant, anti-inflammatory, and anticancer properties [26]. Preclinical studies suggest that rutin can slow tumor growth and cell division [27,28,29,30,31]. However, similar to atorvastatin, it suffers from poor water solubility and rapid metabolism, so a small amount reaches target tissues [24,25,32,33].

Nanocarrier-based delivery systems hold promising advancements to overcome the challenges of traditional drug administration [34]. Among these advancements, liposomes stand out because their phospholipid bilayers can hold both hydrophilic and lipophilic compounds. These carriers help improve drug solubility, protect drugs from premature degradation, and increase bioavailability. Accordingly, liposomal-based systems contribute to reducing unwanted side effects [35,36]. Liposomes can also be designed to deliver drugs to specific sites in the body, potentially boosting treatment effectiveness and reducing harm to healthy tissues [37,38].

Using only one drug to treat cancer is often not enough. Combining drugs can overcome the limits of single agents, improve how well treatment works, and make dosing simpler so patients are more likely to continue their therapy [39]. This is why combining atorvastatin and rutin in a nanoliposome is a logical approach. Atorvastatin promotes cell death and inhibits cell proliferation, while rutin exhibits antioxidant and anti-inflammatory effects. Together, they might be more effective than either drug alone [40]. Adding targeting molecules to the liposome surface could help direct the treatment to where it is needed most.

In this study, we prepared and characterized a nanoliposomal formulation loaded with both atorvastatin and rutin. We tested its physical stability and checked its antioxidant, anti-inflammatory, and selective cytotoxic activity in cell models. This type of system could provide a practical platform for repurposing existing drugs in cancer therapy and potentially in inflammatory diseases.

## 2. Results

### 2.1. Development and Validation of HPLC Method for the Detection of Atorvastatin and Rutin

**Figure 1:**
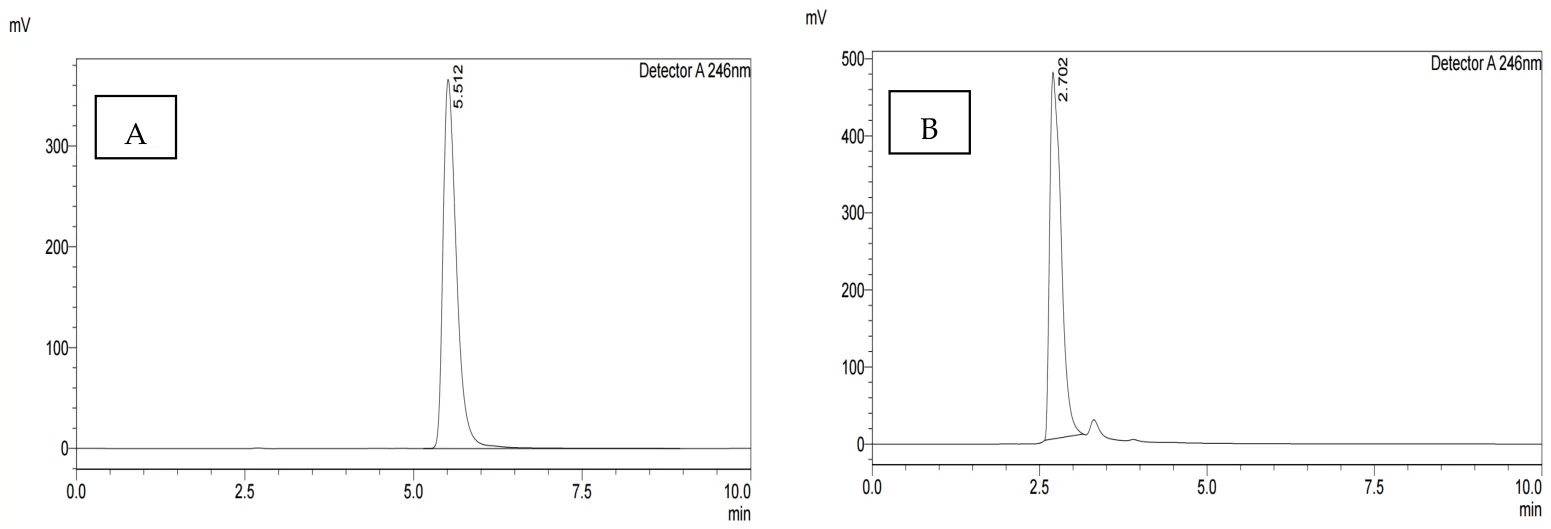
HPLC specificity results: (**A**) Atorvastatin chromatogram with a retention time of 5.512 min; (**B**): Rutin chromatogram with a retention time of 2.702 min.

A reverse-phase HPLC method was successfully developed and validated for the simultaneous detection of atorvastatin and rutin. Method specificity was confirmed by the presence of distinct, well-resolved peaks: atorvastatin at 5.51 min and rutin at 2.702 min. No interfering peaks were observed, indicating high selectivity for both compounds in the co-loaded formulation (Figure 1A,B).

**Figure 2:**
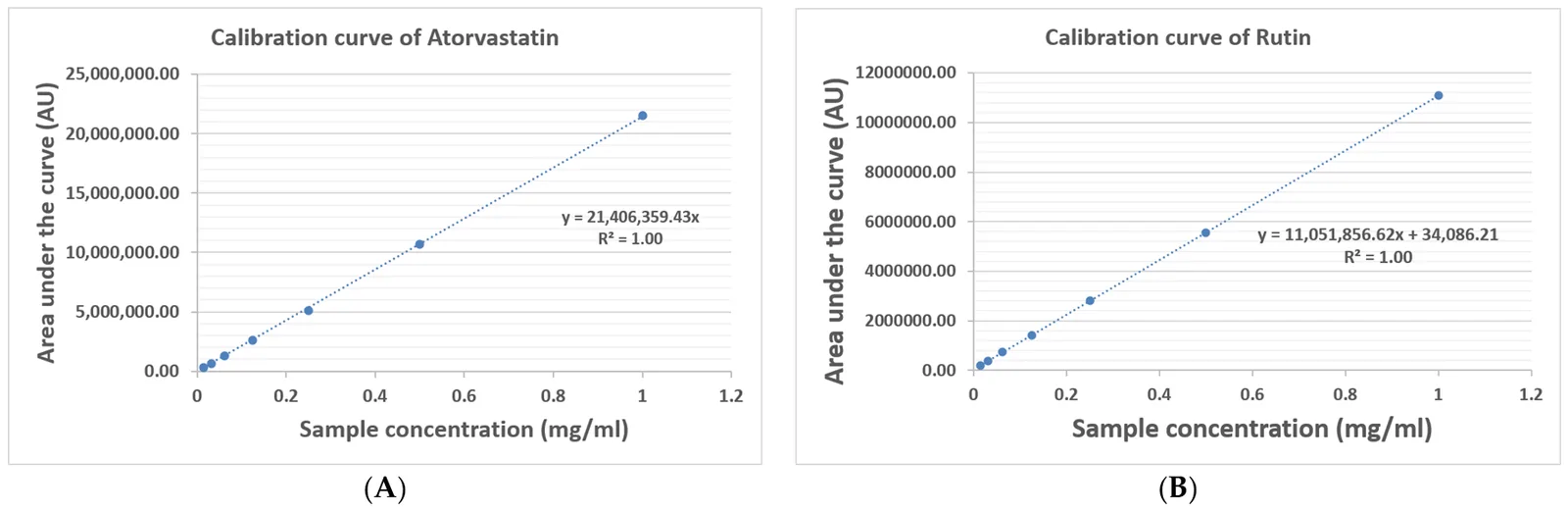
Calibration curves for atorvastatin (**A**) and rutin (**B**) using the validated HPLC method, created by plotting concentration against peak area.

Linearity of the method was evaluated using standard solutions ranging from 0.008 mg/mL to 1 mg/mL. Both compounds exhibited excellent linear correlations between concentration and peak area, with R^2^ values of 0.999 for atorvastatin and 1.000 for rutin. These results confirm that nearly all variation in peak area is attributable to analyte concentration, validating the method’s quantitative reliability. The linear regression equations are presented in Figure 2A,B.

### 2.2. Encapsulation Efficiency and Drug Loading

**Figure 3:**
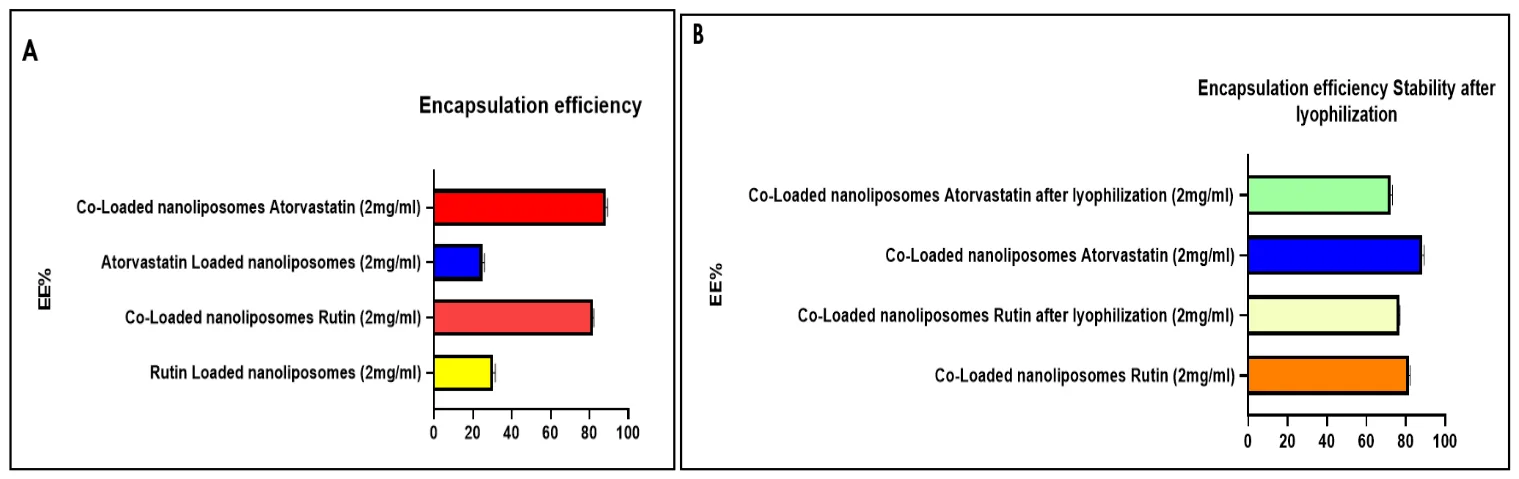
(**A**) Encapsulation efficiency, and (**B**) Stability of encapsulation efficiency after lyophilization.

Encapsulation efficiency data are presented in Figure 3A. The co-loaded liposomes showed substantially higher EE values than the single-drug formulations. For rutin, EE increased from 30.27% ± 1.28% to 81.74% ± 0.44% after co-loading with atorvastatin. Likewise, the EE of atorvastatin increased from 24.90% ± 1.07% to 88.46% ± 0.87%.

The difference between the single- and co-loaded systems was noticeable for both compounds. While the exact mechanism requires further investigation, the data suggest that the presence of both drugs improved their incorporation into the liposomal matrix. Another observation was the lower variability associated with the co-loaded formulation, reflected by the smaller standard deviation values. Collectively, the co-loaded system provided higher drug entrapment and more uniform formulation characteristics than the corresponding single-drug liposomes.

Complementing the EE% data, the Drug Loading capacity (DL%) for the co-loaded nanoliposomes was determined to be 3.54% ± 0.12% for atorvastatin and 3.21% ± 0.09% for rutin. In comparison, the single-loaded liposomes exhibited significantly lower loading capacities (0.91% ± 0.05% for atorvastatin and 1.21% ± 0.07% for rutin), matching the trends observed with encapsulation efficiency.

### 2.3. Stability of Encapsulation Efficiency Following Lyophilization

The stability of EE% following the lyophilization of co-loaded nanoliposomes containing rutin and atorvastatin is shown in Figure 3B. After lyophilization, atorvastatin EE% dropped from 88.46% ± 0.87 to 72.31% ± 0.87. Similarly, rutin’s EE% was 81.74% ± 0.44 at first, but after lyophilization, it slightly decreased to 76.63% ± 0.44.

This small drop in EE% for both materials suggests that freeze-drying may have resulted in minimal surface or structural changes. However, the co-loaded liposomes generally retained a high EE%. This suggests strong formulation stability and long-term storage appropriateness. The formulation’s stability and potential for development as a freeze-dried treatment alternative for clinical applications are highlighted by the consistent medication particle size profile following lyophilization.

### 2.4. Characterization of Nanoliposomes

**Figure 4:**
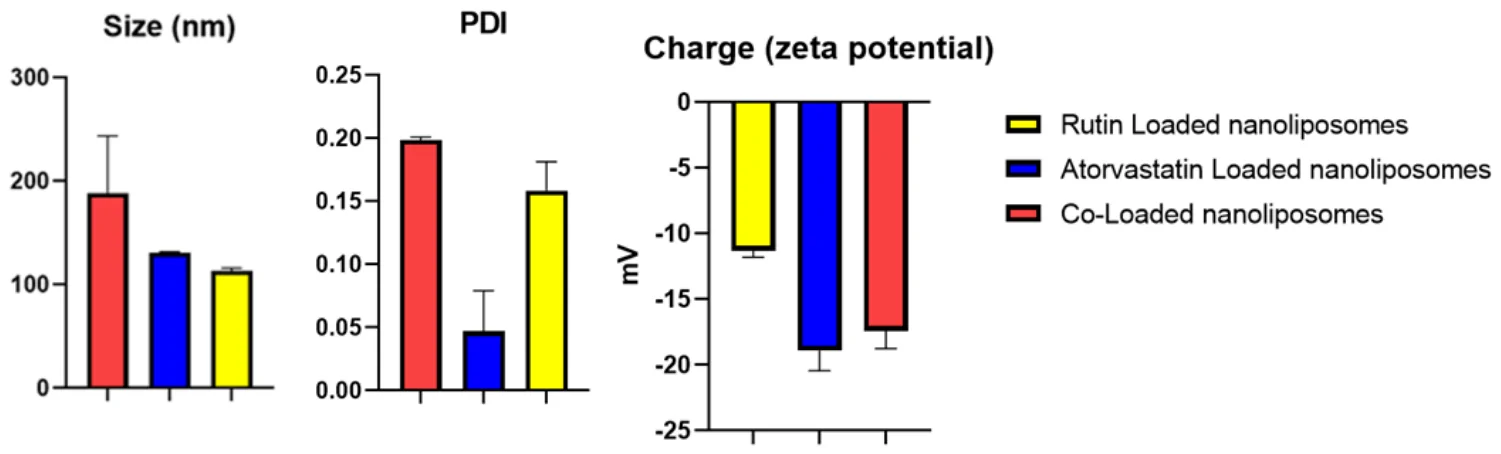
Nanoliposome characterization: particle size (nm), surface charge (mV), and polydispersity index (PDI).

In Figure 4, the particle size characteristics of single-loaded and co-loaded nanoliposome formulations are compared. The nanoliposomes loaded with atorvastatin had an average particle size of 130.7 ± 0.8 nm, a PDI of 0.047 ± 0.032, and a zeta potential of −18.9 ± 1.56 mV. In contrast, the rutin-loaded nanoliposomes had a slightly smaller particle size of 113.37 ± 2.57 nm, a PDI of 0.16 ± 0.02, and a zeta potential of −11.33 ± 0.46 mV. The co-loaded formulation displayed a larger average particle size (188.45 ± 55.15 nm), with a PDI of 0.199 ± 0.003 and a zeta potential of −17.43 ± 1.32 mV. These findings suggest that combining both drugs in the same liposomal structure increases particle size and polydispersity while maintaining a moderately negative surface charge. These factors are crucial in determining the stability of colloidal systems and the release of drugs.

**Figure 5:**
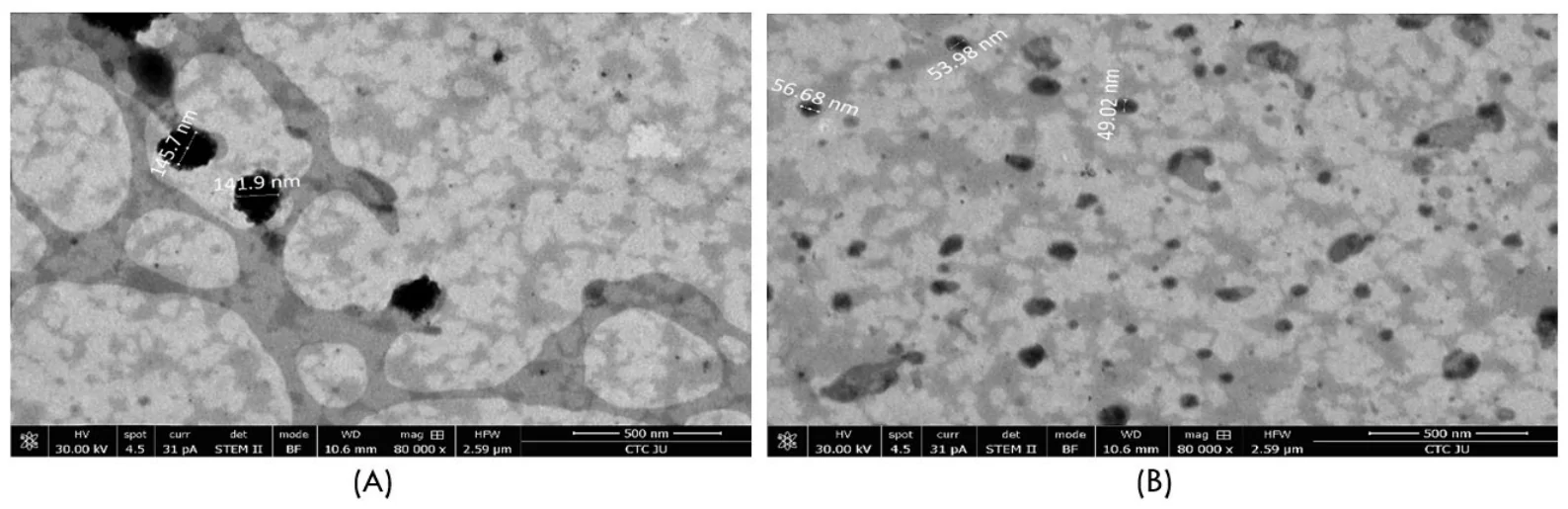
TEM images of nanoliposomes: (**A**) Co-loaded nanoliposomes, (**B)** atorvastatin-loaded nanoliposomes.

TEM analysis verified that the developed liposomes had a spherical shape and were in the nanoscale range (Figure 5A,B). The co-loaded nanoliposomes exhibited distinct vesicle morphologies with consistent sizes, whereas the single atorvastatin-loaded ones appeared smaller and denser. These visual findings aligned with the dynamic light scattering data (Figure 4). This confirms the successful formation of nanoliposomes with a structure and stability suitable for drug delivery.

### 2.5. In Vitro Drug Release Profiles

**Figure 6:**
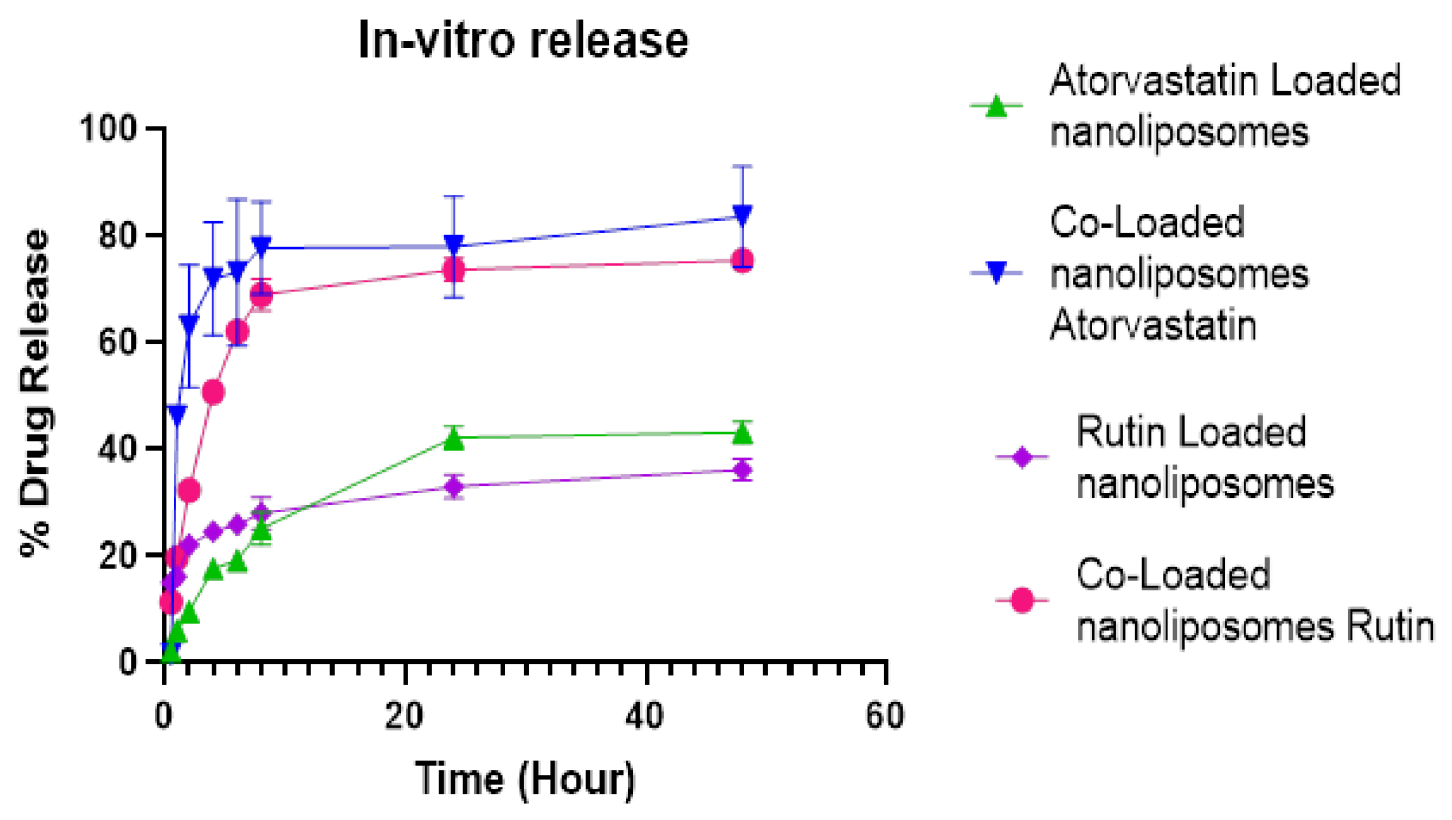
In vitro release of atorvastatin and rutin from single-loaded and co-loaded nanoliposome formulations in phosphate buffer (pH 7.4, 37 °C) over 48 h.

Over 48 h, the release profiles of rutin and atorvastatin from single-loaded and co-loaded nanoliposome formulations were assessed (Figure 6). Cumulative release for liposomes loaded with atorvastatin was 2.17% at 0.5 h, 19.09% at 6 h, and 43.13% at 48 h. On the other hand, atorvastatin showed noticeably greater release in co-loaded liposomes (1.63% at 0.5 h, 73.05% at 6 h, and 83.47% at 48 h). For rutin, a similar scenario happened. The rutin in co-loaded liposomes presented 11.40% at 0.5 h, 62.02% at 6 h, and 75.26% at 48 h, while rutin-loaded liposomes demonstrated 14.95% release at 0.5 h, 25.91% at 6 h, and 36.12% at 48 h.

According to these results, co-loading greatly increases drug release when compared to single-loaded formulations. This indicates faster in vitro release from the co-loaded formulation than from the corresponding single-loaded formulations under the tested phosphate-buffer conditions.

### 2.6. Antioxidant and Anti-Inflammatory Activities (DPPH Assay and LPS-Primed Macrophages)

The antioxidant and immunomodulatory qualities of the nanoliposomal formulations are displayed in Table 1 along with a comparison with common reference agents. While the comparable free-drug forms were essentially inactive, the atorvastatin, rutin, and their co-loaded combination nanoformulations demonstrated substantial DPPH radical scavenging action. The positive control for the assessment of antioxidants was ascorbic acid.

All the nanoliposomal formulations exhibited notable biocompatibility, causing no harm to periodontal ligament (PDL) fibroblasts, unlike cisplatin, which lacked specificity. Both nano-atorvastatin and the co-loaded liposomes showed potent anti-inflammatory effects (IC50 ≤ 1 μM)[^1^6.1] in LPS-stimulated RAW 264.7 macrophages. After 24 h, they were comparable in potency to indomethacin, and after 48 h, they significantly outperformed it. This pattern may be related to formulation-dependent release behavior during incubation; however, the underlying cellular mechanism was not directly assessed. Importantly, SRB assays confirmed that these anti-inflammatory effects were not accompanied by reductions in macrophage viability, suggesting that nitrite inhibition was not accompanied by detectable macrophage cytotoxicity within the tested concentration range.

### 2.7. Cytotoxicity and Safety Assessment

The antiproliferative activity of cisplatin, atorvastatin, nano-atorvastatin, rutin, nano rutin, and co-loaded nanoliposomes (atorvastatin + rutin) was evaluated using the SRB assay on a panel of human cancer cell lines and normal periodontal ligament (PDL) fibroblasts (Table 2 and Table 3). Cisplatin, as the reference chemotherapeutic agent, exhibited potent dose-dependent cytotoxicity across colorectal cancer cell lines (HCT116, HT-29, CACO2, SW480, SW620) and pancreatic PANC1 cells, with IC_50_ values ranging from 0.76 μM to 38.2 μM.

Free rutin and its nanoliposomal form showed negligible antiproliferative effects. In contrast, nano-atorvastatin, its free form, and co-loaded nanoliposomes demonstrated pronounced cytotoxicity against colorectal cancer cell lines, with IC_50_ values between 1.6 μM and 57 μM, comparable to cisplatin. Importantly, all nanoliposomal formulations exhibited high selectivity, showing no cytotoxicity toward normal PDL fibroblasts, confirming their favorable safety profile (Table 2 and Table 3).

In this study, cisplatin showed the expected antiproliferative effect in all tested non-colorectal cancer cell lines. The IC_50_ values were within the low to moderate micromolar range. This indicates that the assay performed as expected. Atorvastatin reduced cell growth in several cancer cell lines, including A375, A549, PC3, MCF7, T47D, MDA-MB-231, HeLa, and U87. However, its activity improved in many of these cells after loading into nanoliposomes. Nano-atorvastatin showed lower IC_50_ values than the free drug in A549, PC3, MCF7, T47D, and U87 cells. In some cases, its effect was similar to or stronger than cisplatin. This suggests that the nanosystem enhances drug uptake and increases its antiproliferative effect.

Rutin alone had a limited effect on cell growth. After nano-encapsulation, its activity increased. This is likely due to better solubility and better contact with the cells. The co-loaded nanoliposomes also kept clear antiproliferative activity in all tested cancer models. Importantly, combining both drugs in one carrier did not reduce their activity.

### 2.8. Suppressions of Human VEGF by a Multiplicity of Antiproliferative Nanoformulation Treatments in Mammary T47D, HeLa Uterine Cervix, and Lung A549 Adherent Tumor Monolayers as Proof of Concept of Molecular Action Mechanism

Table 3b exhibits the dose dependent and cell line dependent increase in human VEGF levels (pg/mL as quantified by ELISA) with decreasing concentrations of antineoplastic apoptogenic cisplatin (in µM) in descending order of potencies in uterine cervix of HeLA> lung A549 and >mammary T47D adherent tumor monolayers. Rutin, however, exerted effectively marginal modulation of human VEGF secretions in the respective panel of cultured tissues of neoplastic disorders. Evidently in a concentration related anti-VEGF targeted strategy, nanoliposomal co-loaded atorvastatin and rutin proved maximally exceeding or comparably effective to antiangiogenesis-related VEGF levels of nanoliposomal atorvastatins. Nanoliposomal rutin were obviously less effective than nanoliposomal atorvastatin but exquisitely maximally exceeding rutin alone in mitigations of human VEGF concentrations over 72 h incubations in each of uterine cervix, lung and breast wells (the same Table 3b).

Overall, the nanoliposomal system improved the performance of atorvastatin and enhanced the effect of rutin. The results show that nanoliposomal formulation altered the in vitro activity profile of the tested compounds and supports further mechanistic evaluation. In Table 4a,b, descriptive fold-changes were calculated by dividing the IC50 values of the indicated treatment pairs. Large descriptive fold differences were observed for the nanoliposomal formulations compared with the corresponding free-drug formulations. At 48 h, the nanoliposomal formulations showed lower IC50 values for nitrite inhibition than the corresponding free-drug formulations, while RAW 264.7 macrophage viability remained within the tested non-cytotoxic range.

Because no formal synergy model, cellular uptake assay, apoptosis assay, cell-cycle analysis, or pathway-specific mechanism assay was performed, the Results are interpreted as comparative in vitro activity profiles rather than evidence of pharmacological synergy or defined molecular mechanism.

### 2.9. Molecular Docking Analysis of Atorvastatin and Rutin with Apoptosis-Related Targets

To explore possible molecular interactions underlying the observed antiproliferative effects, atorvastatin and rutin were docked against selected apoptosis-related proteins, including BCL-2, BCL-XL, MCL-1, caspase-3, and caspase-7. The docking protocol was first evaluated by redocking the corresponding co-crystallized ligands into their binding sites. The reference poses were reproduced within 2 Å for MCL-1 and caspase-7, whereas higher RMSD values were obtained for BCL-2, BCL-XL, and caspase-3. Nevertheless, the predicted poses were located within the experimentally defined binding pockets for all five targets.

#### 2.9.1. Selection and Validation of Protein Structures

The docking protocol was first evaluated by redocking co-crystallized ligands. As shown in Table 5, pose retrieval met the conventional 2.0 Angstrom criterion at MCL-1 and caspase-7 and failed at the other three, which carry the largest and most flexible reference ligands. Repeating the redocking with fourfold expanded conformational sampling did not rescue them, so the limitation lies in ranking the correct pose rather than in generating it. Site recovery is nevertheless good throughout: the top-ranked pose sits in the correct pocket at every target, with its centroid within 1.8 Angstroms of the crystallographic ligand and 73 to 100 per cent of its volume overlapping the experimental pose. The large deviations arise from atom-to-atom correspondence in elongated, pseudo-symmetric ligands, where a reversed placement fills the same groove while displacing every atom.

#### 2.9.2. Docking Performance and Predicted Binding

Atorvastatin and rutin were able to occupy the binding sites of all five proteins, although their predicted scores were generally less favorable than those of the corresponding reference ligands (Table 6). Atorvastatin showed a more favorable score than rutin for BCL-2, MCL-1, caspase-3, and caspase-7, whereas rutin scored slightly better at BCL-XL. The most favorable atorvastatin score was obtained with MCL-1 (−6.51 kcal/mol), while rutin showed its most favorable score with BCL-XL (−6.24 kcal/mol).

The reference ligand scores best at every target, by 3.2 to 5.9 kcal per mole over the better of the two test compounds, except at caspase-3. There the reference is a small 22-heavy-atom covalent fragment whose binding is driven by the bond to Cys163 rather than by non-covalent complementarity, so its non-covalent score of −5.09 is unimpressive and both test compounds edge past it. That is an artifact of scoring a covalent fragment non-covalently, not evidence that either compound binds caspase-3 well. Atorvastatin scores better than rutin at four of the five targets, consistent with the hydrophobic character of these sites and with rutin’s large desolvation penalty.

#### 2.9.3. Binding Interactions and Binding-Site Analysis

The interaction analysis showed different binding patterns for the two compounds. Rutin formed several hydrogen bonds within the examined binding sites, whereas atorvastatin showed fewer hydrogen bonds but more extensive hydrophobic contacts. At MCL-1, both compounds interacted with residues located within the canonical binding groove, including Arg263, Val249, Thr266, and Phe270. At the caspase targets, both compounds occupied the catalytic cleft, but neither was predicted to form a chemically plausible covalent interaction with the catalytic cysteine residues (Table 7).

**Figure 7:**
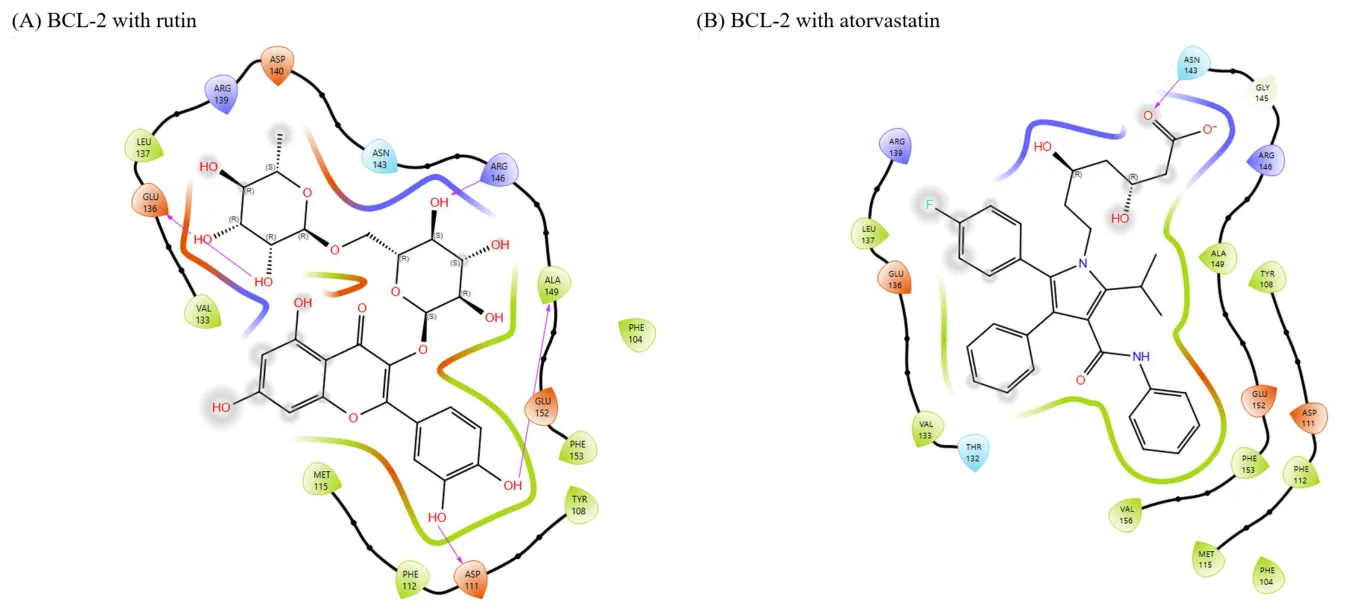
Maestro ligand interaction diagrams for BCL-2: (**A**) rutin and (**B**) atorvastatin, top-ranked pose in each case.

**Figure 8:**
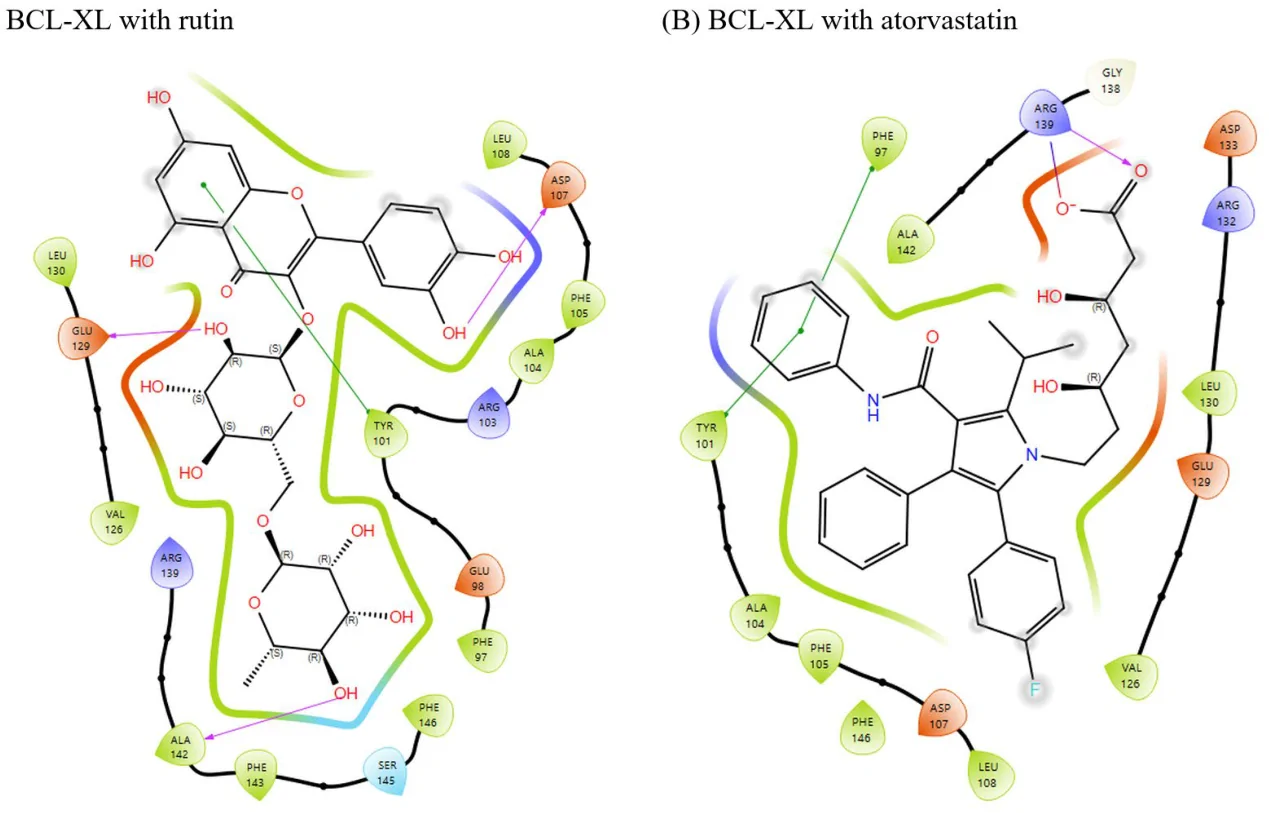
Maestro ligand interaction diagrams for BCL-XL: (**A**) rutin and (**B**) atorvastatin, top-ranked pose in each case.

**Figure 9:**
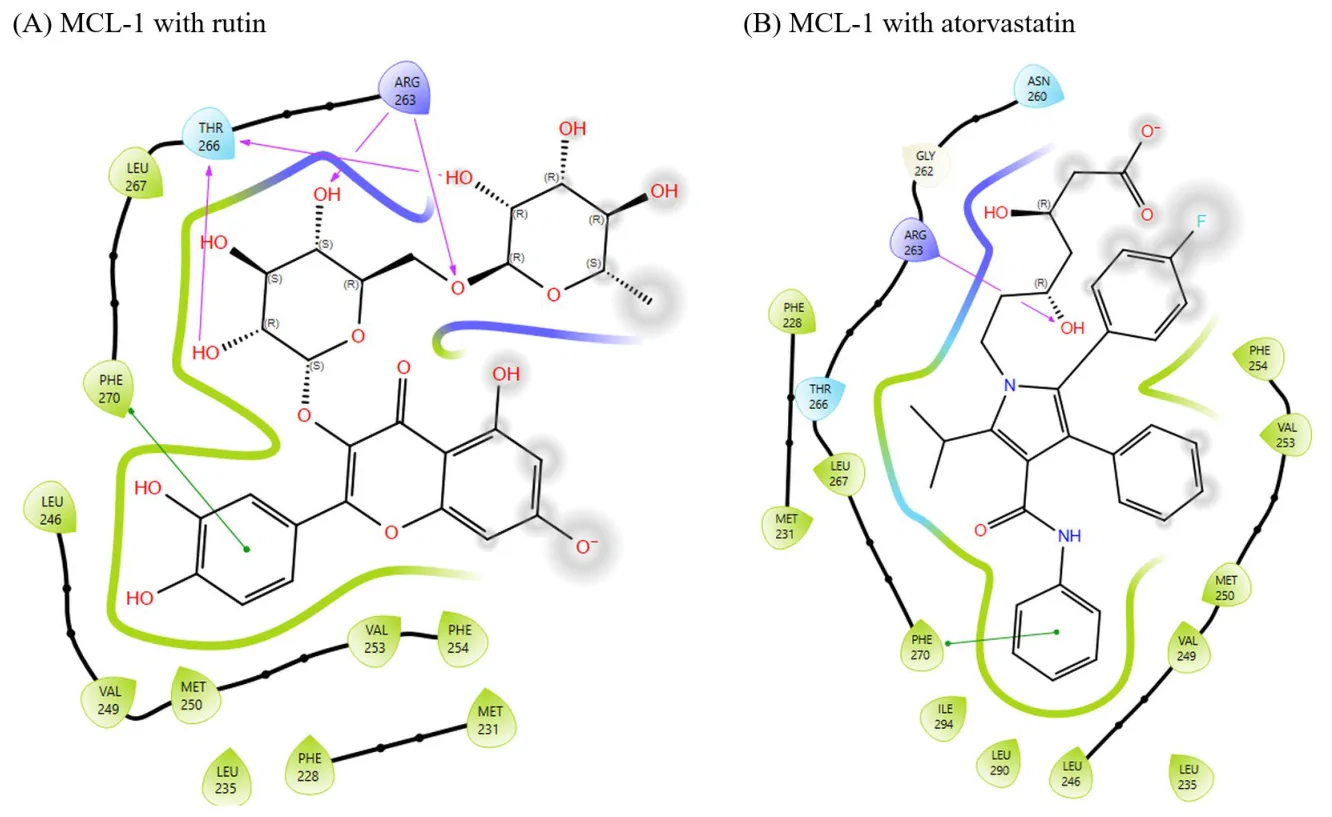
Maestro ligand interaction diagrams for MCL-1: (**A**) rutin and (**B**) atorvastatin, top-ranked pose in each case.

**Figure 10:**
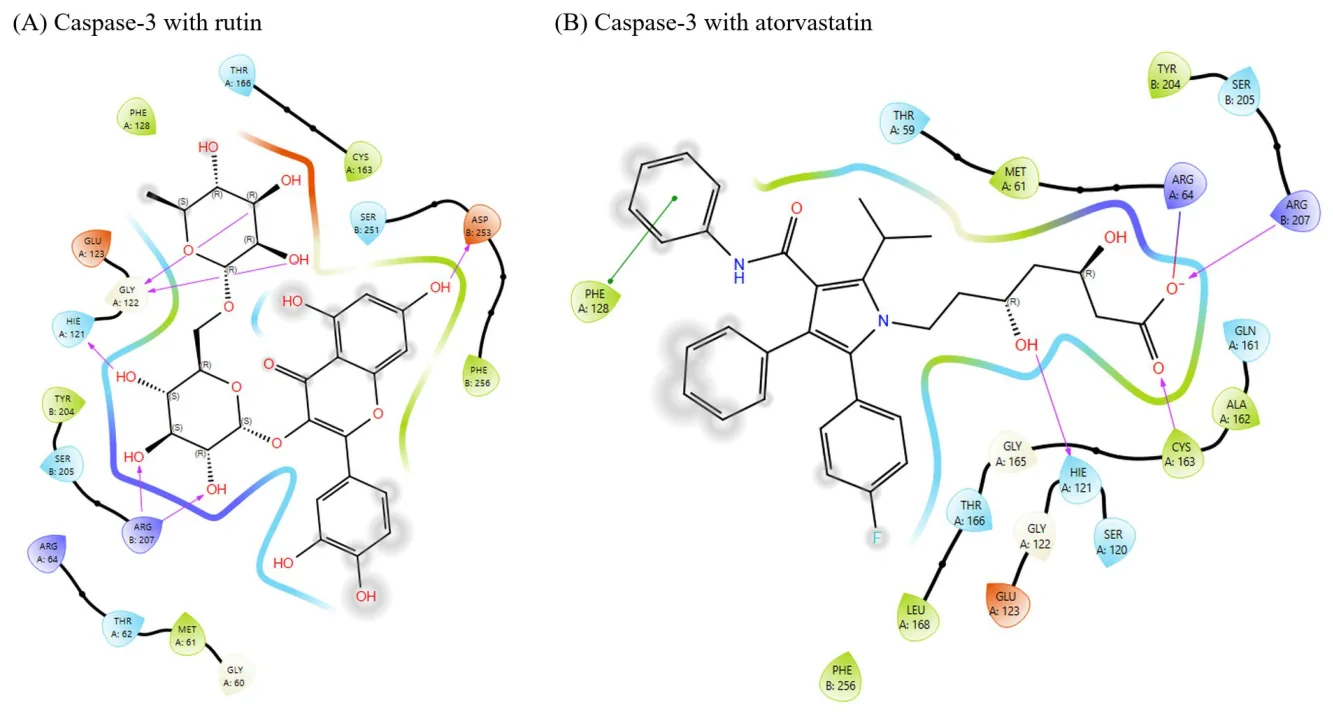
Maestro ligand interaction diagrams for Caspase-3: (**A**) rutin and (**B**) atorvastatin, top-ranked pose in each case.

**Figure 11:**
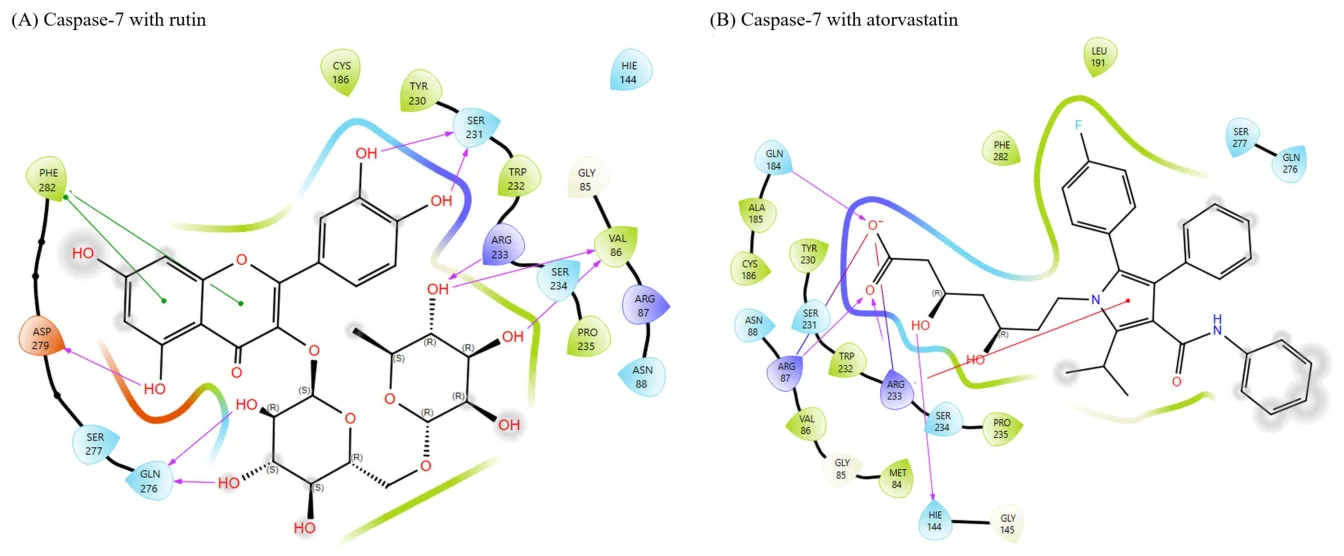
Maestro ligand interaction diagrams for caspase-7: (**A**) rutin and (**B**) atorvastatin, top-ranked pose in each case.

Rutin forms more hydrogen bonds than atorvastatin throughout, four to eight against one to five, as its ten donors and sixteen acceptors predict, while atorvastatin makes more extensive hydrophobic contact and is the only one of the two to show aromatic stacking. Both engage the canonical groove residues of the BCL-2 family, including Phe104, Asp111, Phe112, Arg146 and Phe153 in BCL-2 and Met231, Val249, Arg263 and Phe270 in MCL-1, the positions occupied by the BH3-mimetic inhibitors; in both caspases the poses lie in the catalytic groove adjacent to the Cys and His dyad (Figure 7, Figure 8, Figure 9, Figure 10 and Figure 11).

Hence, both compounds adopt geometrically reasonable poses in all five sites, both score well below the optimized co-crystallized ligands, and neither engages either caspase covalently. They fail the scoring function in opposite and chemically informative ways: rutin pays a large desolvation penalty when its ten donors and 266 square Angstrom polar surface are buried in a hydrophobic groove, while the carboxylate of atorvastatin, anionic at physiological pH, is poorly accommodated by the hydrophobic BH3 groove.

#### 2.9.4. Covalent Docking Assessment

Covalent chemistry behaved as intended, the bond forming at Cys163 and Cys186 in the respective enzymes. Pose ranking repeated the pattern of Table 6: for caspase-7 a pose within 1.11 Angstroms of the crystallographic adduct was generated but ranked nineteenth, and for caspase-3 the best pose reached 4.03 Angstroms at rank two.

Neither test compound forms a chemically credible covalent bond with either caspase (Table 8). Rutin contains no halide and was rejected outright by the nucleophilic substitution chemistry, which reported no matching ligand atoms for all three of its ionization states. Atorvastatin was matched, but only through the fluorine of its 4-fluorophenyl ring, which is its sole halide; an inactivated aryl fluoride of this kind is not displaced by a cysteine thiolate under physiological conditions, so the match is a limitation of the reaction template rather than real chemistry. Rutin can likewise be matched by double-bond addition chemistry through its conjugated flavone carbonyl, with the same objection. Both compounds were therefore treated as non-covalent binders throughout. One genuine caveat deserves a record: flavonols such as rutin and its aglycone quercetin can form Michael adducts with cysteine thiols after oxidation of the B-ring catechol to an ortho-quinone. That route requires a prior oxidation step that docking does not model, and there is no evidence of direct covalent engagement by rutin itself.

## 3. Discussion

Recent advances in nanocarrier technology highlight the potential of vesicular delivery systems to enhance the therapeutic efficacy of repurposed drugs by improving solubility, bioavailability, and targeted delivery, particularly for compounds with antioxidant, anti-inflammatory, and anticancer potential (e.g., micellar or liposomal systems of flavonoids and small molecules). Indeed, nano-micelles of compounds such as thymoquinone, rutin, and capsaicin have demonstrated significantly enhanced antiproliferative, antioxidant, and anti-inflammatory activities compared to their free counterparts [41,42,43,44,45]. Building on this paradigm, our study developed and characterized a liposomal formulation co-loaded with atorvastatin and rutin and evaluated its antioxidant, anti-inflammatory, and SRB-based cytotoxic activity in vitro.

Our co-loaded nanoliposomes achieved high encapsulation efficiencies (88.46% for atorvastatin; 81.74% for rutin) using the thin-film hydration method, confirming the suitability of this technique for dual-agent liposomal systems. Compared to single-agent liposomes, co-loading markedly improved drug entrapment (e.g., rutin’s EE increased from ~30.3% in single loading to ~81.7% in co-loaded form). These findings indicate higher measured encapsulation efficiency in the co-loaded formulation under the tested preparation conditions. Similar results were reported in a study where EE% improved in a multi-phase nanoemulsion co-loaded with hydrophilic arbutin and hydrophobic coumaric acid [46].

The higher in vitro activity observed for several nanoliposomal formulations compared with the corresponding free-drug forms may be related to formulation-dependent changes in dispersion and release. In antioxidant (DPPH) and anti-inflammatory (LPS-stimulated macrophage) assessments, the nanoformulations displayed notably improved performance, suggesting that encapsulation may have altered compound dispersion or assay-accessible drug availability under the tested conditions. In the DPPH assay, which is a cell-free chemical test, the improved activity of the nanoformulations is most likely related to enhanced solubility, improved dispersion, and increased surface availability of the encapsulated compounds rather than cellular uptake.

In the nitrite (NOS) inhibition test, the well-crafted nanoliposomes exhibited efficacy comparable to the standard anti-inflammatory medication, Indomethacin at 24 h and even stronger effects at 48 h, without any minimal harm to normal periodontal ligament fibroblasts (PDL). The enhanced effect observed at 48 h suggests prolonged bioactivity of the nanoformulations under the experimental conditions. These findings support the concept that nanocarriers can improve functional performance in antioxidant and inflammatory models. Overall, these results endorse the concept of co-administering atorvastatin and rutin within a single liposomal system, although additional mechanistic investigations are needed to clarify the mechanisms contributing to the observed enhancement in the effect.

In the nitrite-inhibition assay, the nanoliposomal formulations showed IC_50_ values in the same range as indomethacin at 24 h and lower IC_50_ values at 48 h under the tested conditions. Macrophage viability remained within the tested non-cytotoxic range, indicating that nitrite inhibition was not accompanied by detectable macrophage cytotoxicity. These findings support further evaluation of nanoliposomal atorvastatin and rutin formulations in antioxidant and inflammatory assay systems. Formal synergy modeling was not performed; therefore, the co-loaded formulation should not be interpreted as evidence of pharmacological synergy.

The SRB cytotoxicity results provide an initial in vitro comparison of free-drug, single-loaded, and co-loaded formulations. In most examined cancer cell lines, free rutin and its nanoliposomal form exhibited minimal antiproliferative effect. On the other hand, nano-atorvastatin showed increased cytotoxicity in several models. In certain cancer cell lines, such as prostate (PC3), lung (A549), and glioblastoma (U87), nano-atorvastatin showed IC_50_ values that were either equal to or less than those of the platinum-based medication cisplatin. Additionally, the co-loaded liposomes continued to exhibit detectable antiproliferative action in models of colorectal, skin, lung, prostate, breast, cervical, and glioblastoma. The magnitude of SRB-based growth inhibition varied across cell lines, suggesting cell-line-dependent response patterns.

Furthermore, the nanoliposomal formulations showed no detectable cytotoxicity toward PDL fibroblasts within the tested concentration range, whereas cisplatin reduced PDL fibroblast viability. This finding supports the need for further safety evaluation but should not be interpreted as a complete safety profile [47].

Long-term drug availability is supported by the co-loaded liposomes’ sustained release profile (e.g., around 83.5% cumulative release of atorvastatin at 48 h compared to about 43% for single-loaded liposomes). Although this release pattern suggests formulation-dependent release behavior, pharmacokinetic and in vivo studies are required before conclusions can be drawn regarding exposure duration or therapeutic window.

**Figure 12:**
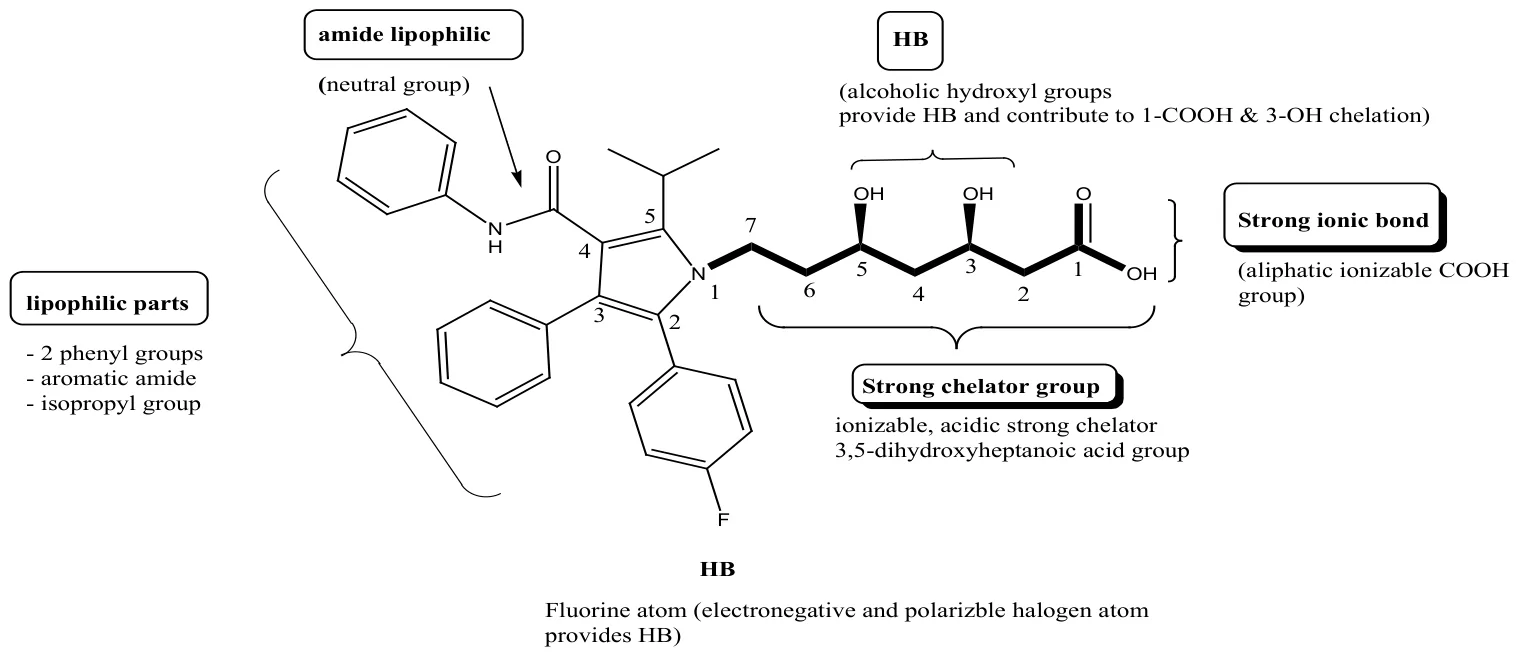
Atorvastatin structure: Underlining main functional groups responsible for binding with nanoliposome.

From a chemical standpoint, the better performance we saw with atorvastatin-loaded nanoliposomes likely comes down to the drug’s own physical and chemical properties—which encapsulation in liposomes seems to amplify [48,49]. Furthermore, the inclusion of drug-loading capacity metrics provides clear boundaries on matrix capability, confirming that co-loading significantly maximizes space utilization within the liposome matrix without jeopardizing structural integrity. Figure 12 shows the structure of atorvastatin, highlighting the main functional groups that help it bind to the liposome. Because atorvastatin is a lipophilic statin, it has a range of pleiotropic and anticancer effects: it blocks the mevalonate pathway, disrupts protein prenylation, modulates YAP/TAZ signaling, triggers apoptosis, and reduces inflammation [50,51]. Its lipophilic nature also means it readily slips into the hydrophobic core of the liposomal bilayer, which makes the bilayer more rigid, cuts down on premature drug leakage, and improves targeted delivery [52]. In our formulation, the moderately negative zeta potential (about −17.4 mV) and polydispersity index (around 0.199) both point to good colloidal stability.

**Figure 13:**
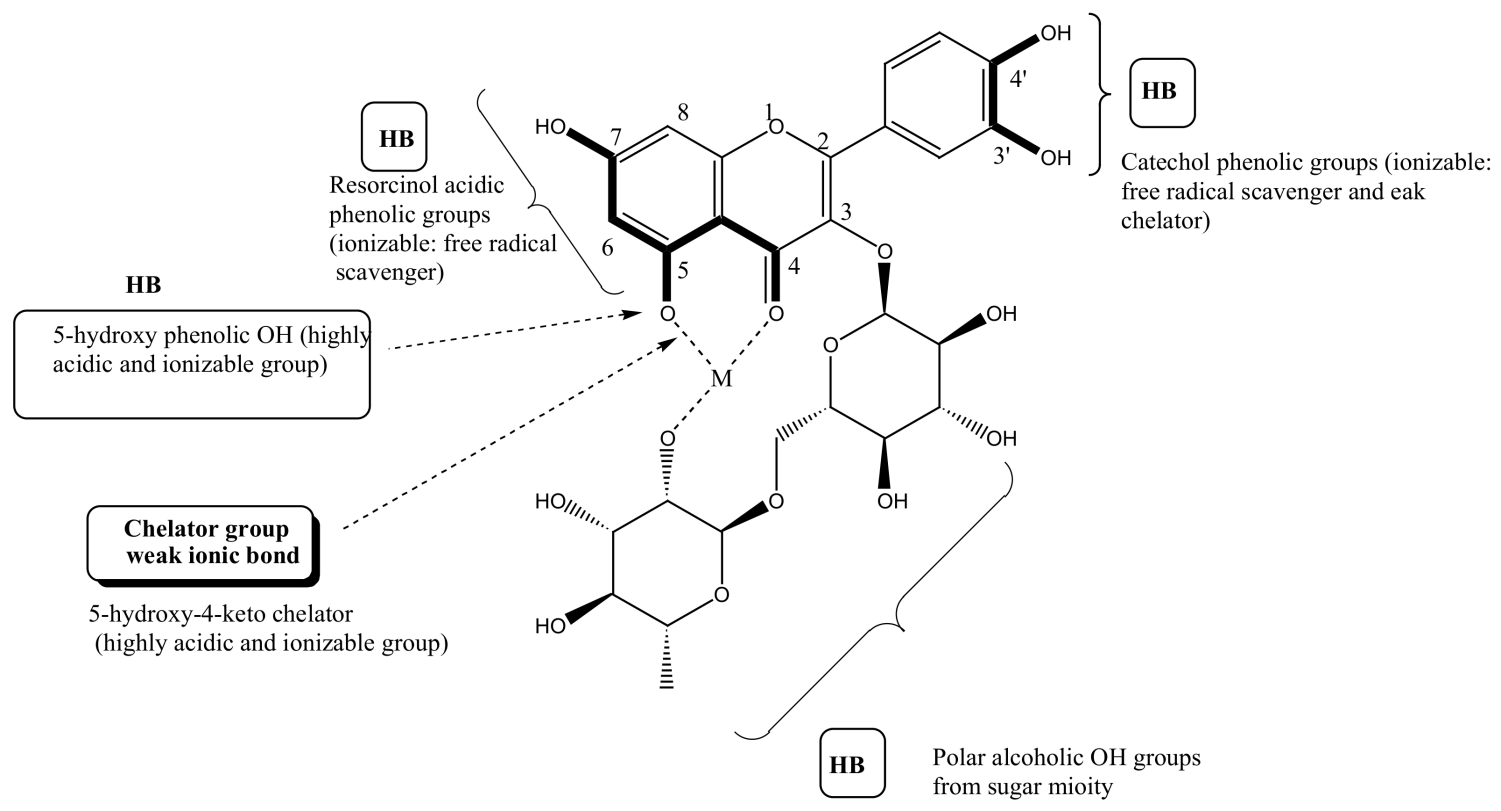
Rutin structure: Underlining main groups responsible for binding with nanoliposome.

Rutin contributes complementary biological effects, including radical-scavenging activity, metal-ion chelation, and modulation of angiogenic and apoptotic pathways, although its poor solubility and limited bioavailability restrict therapeutic use in free form. Incorporation into the liposomal bilayer enhanced rutin’s stability and cellular uptake, improving antioxidant and cytoprotective potential [53,54]. These benefits likely arise from favorable lipid–drug interactions and mutual stabilization within the bilayer when co-loaded with atorvastatin, thereby contributing to the enhanced biological performance of the formulation. Rutin’s chemical structure, highlighting the main groups responsible for binding with the nanoliposome, is shown in Figure 13 [24,55].

In our conclusive remarks and future directives, despite the positive and encouraging data obtained, some limitations should be acknowledged. In vitro, co-loaded nanoliposomes did not produce substantially greater antiproliferative effects than nano-atorvastatin alone across most cancer cell lines. This may reflect competitive interactions within the liposomal bilayer, such as competition for phospholipid head-groups, reduced availability of free atorvastatin, or steric hindrance, limiting intracellular delivery of atorvastatin.

Additionally, while rutin contributes to antioxidant and anti-inflammatory responses, its intrinsic cytotoxicity in these cell lines is weak, so its impact on direct antiproliferative activity is limited. Thus, rather than merely increasing direct cytotoxicity, the dual-agent approach may provide more benefits in vivo via modifying the oxidative and inflammatory tumor microenvironment. This idea is supported by earlier research, which demonstrates that when statins and flavonoids or other phytochemicals are successfully co-delivered, the anticancer efficacy can be increased [50,51,56]. Henceforth comparative biological explorations and assessments of other nanoliposomal co-treatment ratios were not the study objectives, neither were any other statins; like other potent antineoplastic cerivastatin [57], cotreated with natural quercetin [58], promising capsaicin [42], or rutin [53]. It is also worth noting that the current drug release experiments were conducted under physiological conditions (pH 7.4). While these results confirm the sustained release capability of the co-loaded nanoliposomes in a systemic-like environment, future studies should evaluate release kinetics in acidic media (e.g., pH 5.0–6.5) to better simulate the tumor microenvironment and endosomal compartments, which would further enhance the translational relevance of this delivery system.

Furthermore, as our study only examined in vitro tests, in vivo studies are necessary to confirm these results in physiologically relevant settings. Deeper insights into maximizing the formulation’s therapeutic index and balancing its antioxidant, anti-inflammatory, and anticancer actions may be obtained by evaluating various atorvastatin-to-rutin ratios.

The current study highlights the promising antiproliferative efficacy and favorable safety profile of the developed co-loaded nanoliposomes across diverse cancer cell models. While the SRB assay results provide clear evidence of potent and selective cytotoxicity, the underlying molecular mechanisms—such as the induction of apoptosis, cell-cycle arrest, or modulation of intracellular signaling pathways—warrant further investigation. Future research will be directed toward these mechanistic assessments to fully elucidate the pathways involved and to further substantiate the therapeutic mode of action of this co-delivery platform.

The observed therapeutic improvements may potentially be attributed to enhanced cellular uptake and optimized lipid–drug interactions, although these specific mechanisms were not directly investigated in this study. The current data provides a foundation for this interpretation based on the observed pharmacological outcomes, and future work will utilize cellular uptake studies and intracellular drug quantification to substantiate these mechanistic hypotheses.

Distinctly, inflammation plays a critical role in cancer development/progression and contributes to all stages of carcinogenesis [59]. This relationship is evident in the long-term use of non-steroidal anti-inflammatory drugs (NSAIDs), which has been associated with a decreased risk of various cancers [60]. Consequently, our results highlight the possibility of employing nanoliposomal co-delivery to improve the efficacy of repurposed medications with complementary actions. The liposomal combination of atorvastatin and rutin showed improved antioxidant and anti-inflammatory qualities, continuous drug release, and targeted toxicity against a range of human cancer cell lines while preserving normal fibroblasts. These findings support the usefulness of adaptable liposomal platforms in treating intractable disorders where inflammation and oxidative stress are important factors in the growth and spread of tumors. Furthermore, the formulation’s stability after lyophilisation indicates that it is suitable for long-term storage and large-scale manufacture, both of which are necessary for moving on to preclinical stages.

Exceptionally, cerivastatin, among nine evaluated lipophilic statins, demonstrated notable dual bioactivity, exhibiting both cytotoxic and anti-inflammatory effects [61]. Additionally, emodin, a natural phyto-principle, has been shown to suppress inflammation, carcinogenesis, and cancer progression in an animal model of colitis-associated intestinal tumorigenesis [62]. Evidently, oxidative stress is strongly linked to carcinogenesis, influencing tumor growth, survival, and metastasis [63]. Therefore, antioxidants derived from natural and/or synthetic sources may offer promising strategies for antitumor therapeutic interventions [64,65].

Prominently in our relentless pursuit of translational evidence-based repurposing of pharmacologies, markedly exceeding NSAID indomethacin, safe mitigation of anti-LPS (lipopolysaccharide)-induced inflammation in macrophages 264.7 was attributed to optimally antiproliferative statins, namely atorvastatin, simvastatin and lovastatin [57]. In a striking similarity to statins, lipophilic-acid chelating Reduced FQ 4b of superbly dual DPPH-NO (antioxidative—anti-inflammatory) radicals scavenging propensities exerted exceptionally substantial micromolar antiproliferation in colorectal cancer cells with respective antiproliferative IC_50_ values (<50 µM) of HCT116 0.84 < HT29 1.6 < pancreatic PANC1 5.7 < SW620 9.2 vs. robust and classical proapoptogenic antineoplastic cisplatins (*p* value <0.01–0.001; n = 4) [66,67]. Candidly owing to their profound immunomodulation (mostly via suppression of pro-inflammatory cytokines while super-inducing IL2 [68], pro-apoptotic, anti-proliferative and anti-metastatic potentials), repositioning of fluoroquinolones (FQs) into druggable anti-cancer leads seems to be a highly plausible option [69]. Effectively new hydroxamic acid derivatives of FQs displayed appreciably considerable growth inhibition against A549 Lung adenocarcinoma and HCT-116 Colon carcinoma cell lines [70,71]. Cysteinyl leukotrienes, the inflammatory lipid mediators derived from arachidonic acid in macrophages among the rest, bind to cysteinyl leukotriene receptors, notably CysLT1R and CysLT2R. Remarkably elevated expression of CysLT1R has been identified in vastly various human malignancies, including transitional cell carcinoma (TCC) of the bladder, neuroblastomas, and cancers of the brain, prostate, breast, and colorectum. In bridging inflammation and cancer biology, anti-inflammatory/immunomodulatory cysteinyl leukotriene receptor antagonists (LTRAs; Montelukast, Zafirlukast and Pranlukast) reduced cancer risk in a dose-dependent pattern among asthma patients, with a noticeable chemotherapy preventive impact mostly noted in lung, breast, colorectal, and liver malignancies [72,73]. Mechanistically cysteinyl leukotriene receptor antagonists could superbly activate the intrinsic apoptotic pathway, leading to the cleavage of caspases 3 and 9, and cell cycle arrest in neuroblastoma cell lines [74].

To further explore possible molecular mechanisms underlying the observed antiproliferative activity, molecular docking was performed against selected apoptosis-related targets. Similar computational approaches have been used to explore potential molecular targets and provide mechanistic insight into experimentally observed anticancer activity [75,76,77]. The docking analysis provides a possible molecular explanation for the antiproliferative activity observed in the present study [78].

Both atorvastatin and rutin were able to occupy the binding sites of the anti-apoptotic proteins BCL-2, BCL-XL, and MCL-1, which are important regulators of mitochondrial apoptosis, as well as the catalytic clefts of caspase-3 and caspase-7 [79,80]. Atorvastatin showed the most favorable predicted interaction with MCL-1, whereas rutin formed a larger number of hydrogen-bond contacts across the examined targets. These differences are consistent with the distinct physicochemical properties of the two molecules and may help explain their different interaction patterns. The predicted binding of both compounds within apoptosis-related sites is of particular interest in view of the cytotoxic effects observed for the atorvastatin-containing formulations in the present study. However, the docking results should be considered as a mechanistic hypothesis rather than direct evidence of target inhibition. Experimental studies measuring BCL-2 family protein expression, caspase activation, or mitochondrial apoptotic signaling will be required to determine whether these predicted interactions contribute to the observed antiproliferative effects. The present computational analysis therefore extends the biological findings by identifying potential apoptosis-related protein interactions that can be investigated experimentally in future studies.

Taken together, the docking results provide further insight into the possible molecular interactions of atorvastatin and rutin with apoptosis-related targets and may partly explain the antiproliferative effects observed in this study. These findings, together with the antioxidant and anti-inflammatory activities and the enhanced drug delivery achieved through nanoliposomal co-encapsulation, support the potential of this formulation as a combined approach for cancer therapy.

Overall, the present study demonstrates that co-delivery of atorvastatin and rutin in nanoliposomes may offer a way to integrate their complementary pharmacological activities while improving drug incorporation and biological performance. Although further studies are needed to confirm the predicted molecular interactions and elucidate the underlying apoptotic pathways, the findings provide a basis for further investigation of this co-loaded nanoliposomal system as a potentially effective and accessible anticancer strategy.

## 4. Materials and Methods

### 4.1. Materials

Atorvastatin (TCI chemicals, Shanghai, China) was used as the hydrophobic drug. Rutin (Hunan Sunfull Bio-Tech Co., Ltd., Changsha, Hunan, China) was used as the flavonoid active ingredient. HPLC-grade methanol and acetonitrile for HPLC standards and mobile phases were purchased from Sigma-Aldrich, Darmstadt, Germany. All reagents used in antioxidant and cytotoxicity studies are indicated in the main text.

### 4.2. Preparation of Nanoliposomes

All nanoliposomes were made using a well-established method called thin-film hydration, as previously described [58]. During the formulation, hydrogenated soy phosphatidylcholine (HSPC), cholesterol, and a PEGylated phospholipid were mixed at a 65:30:5 molar ratio. This ratio was selected based on a preliminary study. The selected lipids were first weighed in a clean round-bottom flask, then the drugs were added (atorvastatin, rutin or both). All were dissolved in 5 mL of a 2:3 by volume mix of HPLC-grade chloroform and methanol.

To remove the solvent, the flask was placed on a rotary evaporator with the following setting: 90 rpm for 1–2 h, air pressure of 300 ± 50 kPa and temperature 50–60 °C. After that a thin dry film was stuck to the glass. To ensure that no solvent was left, we let the flasks sit with the tops off in a fume hood for thirty more minutes. This was important as the next steps would not work properly if there was still solvent in the flask.

The dried lipid film was hydrated using a phosphate-buffered saline solution, pH 7.4. The mixture was then mixed continuously using a vortex for 30 min, to form more uniform vesicles and this process was helped by heating in a hot water bath. Also, the liposomal suspension was extruded 14 times through a 100 nm polycarbonate membrane, Whatman^®^, using an Avanti Polar Lipids mini extruder (Avanti Polar Lipids, LLC, Alabaster, AL, USA). This was conducted to reduce particle size and achieve a homogeneous, narrow polydispersity index PDI. The resulting nanoliposomes were then spun in a Pico™ 17 Microcentrifuge (Thermo Scientific™, Thermo Fisher Scientific, Waltham, MA, USA) centrifuge at 7000 rpm to remove any free (unencapsulated) active substances. The supernatant liquid, on top which contained the nanoliposomes was collected and stored. It was kept for further investigation (i.e., checking its physical and chemical properties and encapsulation efficiency).

Three different liposomal formulations were created: (i) liposomes loaded with atorvastatin, (ii) liposomes loaded with rutin, and (iii) liposomes loaded with both atorvastatin and rutin (AR). The exact compositions of these formulations are shown in Table 9.

### 4.3. HPLC Method for Atorvastatin and Rutin

High-performance liquid chromatography (HPLC) (Shimadzu Corporation, Kyoto, Japan) was used to quantify atorvastatin and rutin. A reverse-phase C18 column (250 × 4.6 mm) was employed with a mobile phase of 80% aqueous methanol acidified to pH 3.8 using acetic acid. The flow rate was set at 1.25 mL/min, and the column temperature was maintained at 45 °C. Detection was performed at 246 nm (λ_max_). Standard solutions of atorvastatin and rutin (1 mg/mL in acetonitrile) were injected at 10 µL.

### 4.4. Determination of Encapsulation Efficiency (EE%) and Drug-Loading Capacity (DL%)

Encapsulation efficiency was determined by measuring the absorbance of supernatants after centrifugation. Supernatants were diluted with acetonitrile (1:4), centrifuged for 10 min, and the absorbance was recorded. EE% was calculated using the following formula [81]:

(1) EE% = [(Total drug − Free drug)/Total drug] × 100%

The drug-loading capacity (DL%) was calculated to determine the percentage of active ingredients entrapped relative to the total lipid mass using the following formula:

(2) DL% = [(Total drug − Free drug)/Total lipids] × 100%

### 4.5. Characterization of Nanoliposomes

Particle size, polydispersity index (PDI), and zeta potential were measured using a dynamic light scattering Zetasizer (Malvern Pananalytical Ltd., Malvern, UK). Samples were diluted in deionized water (one part sample to twenty parts water) and analyzed in triplicate. When the PDI value is low, it means the liposomal platform is more uniform, which is necessary for drug delivery [58]. The nanoliposomal formulation was examined using a FEI Morgagni 268D transmission electron microscope (TEM) (Hillsboro, OR, USA). First, a suspension was created with a concentration of approximately 0.05 mg/mL in deionized water. Then, a 10 µL drop of this suspension was placed on a copper grid of 200 mesh (SPI Supplies, West Chester, PA, USA) and allowed to settle for a few minutes to allow the nanoparticles to stick. Any excess liquid was gently removed with filter paper before capturing images. Subsequently, the grids were examined using a Versa 3D transmission electron microscope (TEM) operated at 30 kV (FEI, Eindhoven, The Netherlands). The acquired TEM images were then analyzed and processed using ImageJ^®^ software (version 1.48q; National Institutes of Health, Bethesda, MD, USA). 

### 4.6. Stability Study

The stability study of the developed nanoliposomes was assessed after being stored at −70 °C and lyophilized to complete dryness. Post-lyophilization, EE% was re-evaluated under the same conditions.

### 4.7. In Vitro Drug Release

The release of atorvastatin and rutin from all three nanoliposome formulations was examined using the dialysis bag method. The release was checked at these points: 0.5 h, 1 h, 2 h, 4 h, 6 h, 24 h, 48 h and 72 h. For each formulation of the nanoliposome, 2 mL of the nanoliposome suspension was sealed inside a cellulose ester membrane (molecular weight cut-off: 12,000–14,000 Da). To maintain sink conditions and guarantee uniform dispersion, the sealed dialysis bags were submerged in 50 mL of phosphate-buffered saline (PBS, pH 7.4) and incubated at 37 ± 0.5 °C in a shaking water bath at 100 rpm. To maintain a consistent volume and sink conditions, 1 mL of the external release medium was removed at each measurement point and promptly replaced with an equivalent volume of preheated PBS. The withdrawn samples were then analyzed by HPLC using the same method described earlier [40,82].

The percentage of drug released over time was calculated using the formula:

(3) Release% = [Amount release at t/Total encapsulated drug] × 100%

### 4.8. DPPH-Free Radical Scavenging Assay

This method depends on the reduction in the radicals resulting in a color change from oxidized purple to reduced yellow. Principally DPPH radical is stable and can provide reproducible spectroscopic values. Diphenyl-2-picryl-hydrazyl (DPPH) undergoes reduction in methanol (MeOH) solution, in the presence of a hydrogen-donating compound due to the formation of the non-radical form DPPH-H. Basically, a DPPH solution (0.2 mM) was diluted with MeOH and then mixed with test compounds or ascorbic acid in a concentration ratio of 1:1 using a 96-well plate spanning 6.25–200 µM dose range. Ascorbic acid was the robust and classical standard radical scavenging reference agent for comparison purposes. The treated solution was treated for one hour and isolated from light. Finally, a change in absorbance at 517 nm wavelength was measured using a microplate reader (Bio-Tek Instrument, USA).

The calculation of the DPPH radical scavenging activity inhibition was determined by the following equation, where A represents photometric absorbance: in % = (A control − A sample)/A control × 100%.

After generating a dose–response curve, a linear regression is used to calculate the exact concentration where 50% radical scavenging is achieved. A quadruplet plate assay approach was performed, and the calculated antioxidative activities were reported as the mean IC_50_ value of treatments ± SD (n = 4 independent observations) [83,84,85,86].

### 4.9. In Vitro Physiologically Regulated Anti-Inflammatory Assay

RAW 264.7 murine macrophages (ATCC^®^ TIB-71; (ATCC Manassas, VA, USA)) were cultured in high-glucose DMEM supplemented with 10% FBS, penicillin (100 U/mL), streptomycin (100 μg/mL), and L-glutamate (100 μg/mL) at 37 °C in 5% CO_2_. Lipopolysaccharide (LPS; Sigma, St. Louis, MO, USA, Burlington, MA, USA) was added to confluent macrophages (2 × 10^5^ cells/well) in 96-well plates, followed by treatment with test compounds or indomethacin (5–200 µM). Nitrite levels were measured using Griess reagent, with absorbance read at 550 nm (Spectro Scan, Sedico Ltd., Nicosia, Cyprus). Nitrite concentrations were calculated using a sodium nitrite standard curve. After generating a dose–response curve, a linear regression is used to calculate the exact concentration where 50% immunomodulation/antiinflammation is achieved. IC_50_ values ± SD were calculated from triplicate–quadruplet assays. Cell viability was assessed using the SRB assay to confirm physiologically regulated anti-inflammatory effects [57,87].

### 4.10. Selective Antiproliferation Assays and Suppressions of VEGF by a Multiplicity of Antiproliferative Nanoformulations Treatment in HeLa Uterine Cervix, Mammary T47D and Lung A549 Adherent Tumor Monolayers as Proof of Concept of Molecular Action Mechanism

Cytotoxicity was evaluated using the SRB assay in 96-well plates seeded with various human cancer cell lines: MCF7, T47D, HT-29, HCT116, SW480, SW620, CACO2, PANC1, PC3, A375, A549, HeLa, U87, and MDA-MB-231 (ATCC). Periodontal ligament fibroblasts (PDL; ScienCell, Carlsbad, CA, USA) were used for selectivity assessment. Cells were cultured in high-glucose DMEM (Bio Whittaker, Verviers, Belgium) supplemented with 10% FBS, HEPES buffer (10 mM), L-glutamine (2 mM), gentamicin (50 µg/mL), penicillin (100 U/mL), and streptomycin sulfate (100 mg/mL). Test compounds and cisplatin (reference agent) were applied at concentrations of 3.125–200 µM. After incubation, SRB staining was performed, and absorbance was measured (Spectro Scan, Sedico Ltd., Cyprus). Subsequent to generating a dose–response curve, a linear regression is used to calculate the exact concentration where 50% antiproliferation is achieved and IC_50_ values ± SD (n = 3) were calculated [57,88,89,90,91,92,93,94,95,96]. All three nanoliposomal treatments (at different concentrations) were assessed for suppressing VEGF secretions and subsequent inhibitions of tumor neovascularisation in uterine cervix HeLA, lung A549 and mammary T47D adherent monolayers. Equally both cisplatin and rutin were enrolled for control comparisons of targeted-VEGF cancer therapy strategies. Harvesting of supernatants and their subsequent ABCAM ELISA of quantifications of human VEGF (pg/mL) according to manufacturer instructions were implemented (Waltham, MA, USA). Results were means ± SD (n = 2).

### 4.11. Molecular Docking Studies

#### 4.11.1. Structures and Preparation

One hollo crystal structure was used per target, selected for a genuine inhibitor in the pharmacologically relevant site, the best available resolution, and a wild-type binding-site sequence. Every residue within 5 Angstroms of the reference ligand was compared with the UniProt canonical sequence and matched at all positions. Widely cited alternatives were rejected on inspection: 4LVT contains navitoclax rather than venetoclax, 5LOF is a maltose-binding-protein fusion, and 1NME contains disulfide-tethered fragments. A single biological copy was retained per entry, and in 600K, where venetoclax is modeled in two alternate conformations, only the higher-occupancy conformer was kept. Water and crystallization additives were deleted. Receptors were processed with the Protein Preparation Wizard (Release 2025-1, Schrödinger, LLC, New York, NY, USA; accessed 25 August 2026), with protonation assigned by Epik and PROPKA 3.0. at pH 7.4 and restrained minimization under OPLS4 to 0.30 Angstroms [78].

#### 4.11.2. Ligands, Grids and Docking

Rutin and atorvastatin were built from PubChem isomeric SMILES and prepared with LigPrep and Epik (Release 2025-1, Schrödinger, LLC, New York, NY, USA; accessed 25 August 2026), at pH 7.4 using OPLS4, with stereochemistry preserved rather than enumerated. Preservation was verified by regenerating the standard chemical identifier of every output state, which returned the reference values for both compounds. Grids were centered on the measured centroid of the retained reference-ligand copy, with the box sized from the measured extent of that ligand and of an extended rutin conformer rather than from the default, because the largest reference ligands span close to 20 Angstroms. All dockings used Glide standard precision with flexible conformer generation and post-docking minimization. Pose retrieval was measured as a heavy-atom root-mean-square deviation computed in place, without superposition, with symmetry-equivalent atom mapping, against the deposited crystallographic coordinates rather than the minimized copy.

#### 4.11.3. Covalent Inhibitors and Covalent Docking

Two of the five reference ligands are covalent. In caspase-3 entry 2XYG the inhibitor CAS329306 is a chloromethylketone whose chloride has already been displaced by the catalytic Cys163 thiolate; the deposited model therefore contains the 22-heavy-atom adduct, and the crystallographic LINK record specifies a 1.76 Angstrom bond from Cys163 SG to ligand atom C22. In caspase-7 entry 1F1J the inhibitor acetyl-Asp-Glu-Val-Asp aldehyde forms a hemi thioacetal with Cys186, with a deposited bond length of 1.80 Angstroms. For covalent docking each ligand was rebuilt from SMILES in its unreacted form so that the warhead was intact, since the deposited coordinates represent the product. Caspase-3 used the nucleophilic substitution reaction with chloride as leaving group, and caspase-7 used nucleophilic addition to the carbonyl double bond. Covalent docking was run in enrichment mode with the affinity score and the final Glide stage pinned to standard precision so that the protocol matched the rest of the study. Covalent and non-covalent scores are on different scales and are reported in separate tables.

### 4.12. Statistical Analysis

The values were presented as mean ± SD of 3–4 independent experiments. Statistical differences between reference agent and different treatment drugs were determined using GraphPad Prism software ANOVA test (version 8.0.1 for Windows; GraphPad software, San Diego, CA, USA). Values were considered significantly different if *p* < 0.05, and not significantly (NS) different if *p* > 0.05.

## 5. Conclusions

This study successfully developed and characterized nanoliposomes co-loaded with rutin and atorvastatin. Under the studied conditions, the formulations demonstrated a regulated release profile and good encapsulation efficiency. When compared to the free compounds, in vitro tests showed increased inhibition of nitric oxide generation in LPS-stimulated macrophages and improved antioxidant activity in the DPPH model.

When compared to its free form, nano-atorvastatin demonstrated enhanced antiproliferative activity in several cancer cell lines in cytotoxicity experiments. Measurable cytotoxic effects were sustained by the co-loaded formula in a variety of tumor types. Crucially, normal periodontal ligament fibroblasts showed no discernible cytotoxicity at the studied concentrations, indicating a good in vitro safety profile in comparison to cisplatin. Additionally, the formulations maintained their stability following lyophilization, supporting their potential for preservation and future development.

Overall, these results suggest that atorvastatin and rutin can work better in vitro when co-delivered via nanoliposomes. Although the results are promising, more mechanistic research and in vivo studies are needed to establish therapeutic relevance, pharmacokinetic behavior, and safety. This should be considered before clinical translation can be taken into consideration.

## Figures and Tables

**Table 1:** IC_50_ values (μM) for DPPH radical scavenging activity compared to ascorbic acid, and anti-inflammatory effects in LPS-stimulated RAW 264.7 macrophages compared to indomethacin. Safety assessment shows absence of cytotoxicity against normal periodontal ligament fibroblasts relative to cisplatin.

Treatment	DPPH- IC_50_ Value μM	iNOS- IC_50_ Value μM (24 h)	iNOS- IC_50_ Value μM (48 h)	RAW 264.7 ViabilityIC_50 values_	PDL Fibroblasts IC_50_ Value
Reference Drug	Ascorbic Acid	Indomethacin			Cisplatin
	0.01 ± 0.0	0.028 ± 0.004	0.05 ± 0.01	NI	5.59 ± 0.53
Atorvastatin	164.40 ± 28.28 *	0.465 ± 0.007 *	0.1235 ± 0.004 *	NI	NI
Nano atorvastain	0.06 ± 0.01 NS	0.021 ± 0.001 NS	0.0185 ± 0.0021 NS	NI	NI
Rutin	65.20 ± 12.85 *	0.098 ± 0.01 *	0.058 ± 0.011 NS	NI	NI
Nano Rutin	0.29 ± 0.06 NS	0.100 ± 0.021 *	0.015 ± 0.0004 NS	NI	NI
Nano (Atorvastatin + rutin)	0.01 ± 0.0 NS	0.087 ± 0.003 *	0.0022 ± 0.0003 NS	NI	NI

Results are mean ± SD (n = 3 independent replicates). IC_50_ values (μM) (concentration at which 50% DPPH reduction or NOS inhibition in comparison to non-induced basal incubations) were calculated within testing dose range. NI: Noninhibitory within testing dose range. NI is noninhibitory over the testing dose range. *p*-value calculated by ANOVA test between test compound IC50 values and references’ (μM) using Graph Pad Prism software 8. * When *p* < 0.05 of significant variances and NS: not significantly different from reference agent, when *p* > 0.05. iNOS is inducible Nitric Oxide Synthase; DPPH: 2,2-diphenyl-1-picrylhydrazyl.

**Table 2:** Cytotoxicity (as of %untreated control) IC_50_ value in µM of the tested treatments vs. cisplatin against gastrointestinal and colorectal cancer cells.

Treatment	HCT116	HT29	CACO2	SW480	SW620	PANC1
Cisplatin	13.59 ± 0.4	0.76 ± 0.14	3.61 ± 0.69	1.93 ± 0.25	4.11 ± 0.67	38.20 ± 0.88
Atorvastatin	35.79 ± 1.9 *	39.14 ± 7.02 *	34.27 ± 0.10 *	33.44 ± 0.74 *	1.58 ± 0.23 NS	37.48 ± 1.55 NS
Nano atorvastain	29.81 ± 0.01 *	40.88 ± 0.34 *	14.37 ± 2.94 *	28.67 ± 0.67 *	43.45 ± 0.07*	35.79 ± 0.30 NS
Rutin	154.70 ± 1.06 *	136.44 ± 3.2 *	67.17 ± 1.13 *	147.20 ± 9.51 *	247.17 ± 22.12 *	140.24 ± 0.88 *
Nano rutin	59.05 ± 3.13 *	67.07 ± 2.30 *	34.79 ± 5.57 *	90.66 ± 0.34 *	67.66 ± 3.46 *	92.90 ± 3.34 *
Nano (atorvastatin + rutin)	43.36 ± 1.28*	41.23 ± 0.50 *	45.53 ± 2.17 *	37.25 ± 0.76 *	57.13 ± 2.44 *	105.00 ± 6.22 *

Results are mean ± SD (n = 3–4 independent replicates). IC_50_ values (concentration at which 50% inhibition of cell proliferation took place in comparison to non-induced basal 72 h incubations) were calculated within 3.125–200 µM range. *p*-value calculated by ANOVA test between test compound IC50 values and references’ (μM) using Graph Pad Prism software 8. * When *p* < 0.05 of significant variances and NS is not statistically significantly different from reference agent, when *p* > 0.05.

**Table 3:** (**a**) Cytotoxicity (as of %untreated control) IC_50_ value in µM of the tested treatments vs. cisplatin against other cancer cell lines. (**b**) Mitigation of human VEGF by a multiplicity of antiproliferative nanoformulation treatments in HeLa uterine cervix, mammary T47D and lung A549 adherent tumor monolayers.

(**a**)						
**Cancer Cell Line**	**Cisplatin**	**Atorvastatin**	**Nano-Atorvastatin**	**Rutin**	**Nano Rutin**	**Nano (Atorvastatin + Rutin)**
A375 (Skin melanoma)	22.72 ± 1.23	68.96 ± 2.64 *	57.84 ± 3.54 *	187.82 ± 1.40 *	87.75 ± 3.68 *	110.60 ± 2.82 *
A549 (Lung cancer)	33.03 ± 2.49	23.09 ± 0.66 *	16.74 ± 0.75 *	59.25 ± 2.24 *	35.98 ± 2.06 NS	38.20 ± 1.26 *
PC3 (Prostate cancer)	12.67 ± 0.16	7.01 ± 1.25 NS	4.77 ± 0.10 NS	243.13 ± 0.81 *	157.26 ± 21.49 *	89.21 ± 7.79 *
MCF7 (Mammary cancer)	28.71 ± 1.83	40.87 ± 1.21 *	31.13 ± 0.19 NS	126.38 ± 6.25 *	84.07 ± 5.99 *	66.76 ± 3.51 *
T47D (Mammary cancer)	32.12 ± 0.40	34.58 ± 0.60 NS	29.12 ± 0.02 NS	142.42 ± 0.74 *	74.23 ± 8.02 *	50.71 ± 3.24 *
MDA-MB-231 (Triple-ve)	12.92 ± 2.52	32.58 ± 0.00 *	35.85 ± 1.04 *	143.04 ± 5.58 *	117.55 ± 4.68 *	57.27 ± 1.12 *
HeLa (Cervix cancer)	27.75 ± 1.72	38.06 ± 1.22 *	35.58 ± 1.52 *	175.44 ± 2.20 *	79.82 ± 3.10 *	105.35 ± 1.29 *
U87 (Glioblastoma)	15.76 ± 2.78	15.00 ± 0.83 NS	10.39 ± 1.56 *	109.90 ± 2.05 *	67.65 ± 5.20 *	35.65 ± 0.07 *
(**b**)						
**Treatments/Tumor Cell Lines**	**HeLa Uterine Cervix Tumerogenesis**		**A549 Lung Tumerogenesis**		**T47D Mammary Tumerogenesis**	
Human VEGF (pg/mL)						
Nano-Atorvastatin (mg/mL)						
0.396	16.5 ± 0.71		23 ± 3.5		15.5 ± 0.7	
0.198	385 ± 57.75		313.5 ± 47		30 ± 5.7	
0.099	812 ± 121.8		474.5 ± 27.6		138.5 ± 19.1	
Nano Rutin (mg/mL)						
0.24	182 ± 4.24		160.5 ± 7.8		124 ± 14.1	
0.12	842 ± 12.7		1108.5 ± 30.4		190 ± 26.9	
0.06	833.5 ± 4.95		1168.5 ± 23.3		235 ± 2.8	
Co-Loaded Nanoatrovastatin and Rutin (mg/mL)						
0.143 Rutin + 0.335 Atorvastatin	20.5 ± 0.71		20.5 ± 3.1		17 ± 0.003	
0.0715 + 0.1675	425 ± 15.56		530.5 ± 91.2		73.5 ± 3.5	
0.036+ 0.084	642 ± 33.94		649.5 ± 21.9		214.5 ± 4.9	
Rutin (µM)						
200	678 ± 4.24		820 ± 26.9		288 ± 28.3	
100	802 ± 35.36		1084.5 ± 40.3		316 ± 11.3	
50	842.5 ± 23.33		1173 ± 89.1		411.25 ± 52	
Cisplatin (µM)						
200	27 ± 1.41		32 ± 1.4		53.5 ± 3.5	
100	48 ± 0.001		56 ± 0.03		221 ± 7.1	
50	67.5 ± 3.54		84 ± 12.6		555 ± 83.3	

Results are mean ± SD (n = 3–4 independent replicates). IC_50_ values (concentration at which 50% inhibition of cell proliferation took place in comparison to non-induced basal 72 h incubations) were calculated within 3.125–200 µM range. *p*-value calculated by ANOVA test between test compound IC50 values and references’ (μM) using Graph Pad Prism software 8. * When *p* < 0.05 of significant variances and NS is not statistically significantly different from reference agent, when *p* > 0.05.

**Table 4:** (**a**) Co-loaded nanoliposomes of atorvastatin and rutin: A nanocarrier strategy for chemosensitisation of repurposed antioxidation pharmacologies. (**b**). Co-loaded nanoliposomes of atorvastatin and rutin: A nano-carrier strategy for chemosensitisation of repurposed anti-inflammatory pharmacologies.

(**a**)				
**Repurposed Pharmacologies of Antioxidation**	**Fold Change in Repurposed Pharmacologies**			
Atorvastatin nanoliposomes vs. atorvastatin alone	2680.4× folds potentiating of antioxidative efficacies		Effective chemosensitisation of atorvastatin capacities	
Rutin nanoliposomes vs. rutin alone	228.75× folds potentiating of antioxidative efficacies		Effective chemosensitisation of Rutin capacities	
Atorvastatin–rutin co-loaded nanoliposomes vs. nanoliposomes of atorvastatin	9.7× folds potentiating of antioxidative efficacies		Effective chemosensitisation of atorvastatin nanoliposomal capacities	
Atorvastatin–rutin co-loaded nanoliposomes vs. nanoliposomes of rutin	45.2× folds potentiating of antioxidative efficacies		Effective chemosensitisation of Rutin nanoliposomal capacities	
Atorvastatin–rutin co-loaded nanoliposomes vs. atorvastatin	26,095.2× folds potentiating of antioxidative efficacies		Effective chemosensitisation of atorvastatin capacities	
Atorvastatin–rutin co-loaded nanoliposomes vs. rutin	10,348.4× folds potentiating of antioxidative efficacies		Effective chemosensitisation of Rutin capacities	
(**b**)				
**Repurposed Pharmacologies of Physiologically Regulated Anti-Inflammation/Immunomodulation**	**24 h Sustained Release**		**48 h Sustained Release**	
	**Fold Change in Repurposed Pharmacologies**		**Fold Change in Repurposed Pharmacologies**	
Atorvastatin nanoliposomes vs. atorvastatin alone	22× fold augmentation of potencies	Effective chemosensitisation of atorvastatin capacities	6.7× fold enhancement of atorvastatin potencies	Sustained potentiating of atorvastatin pharmacologies
Rutin nanoliposomes vs. rutin alone	No significant discrepancies in efficacy	Lack of potentiating of affinities	3.9× fold augmentation	Newly emerging potentiating of rutin effects
Atorvastatin–rutin co-loaded nanoliposomes vs. nanoliposomes of atorvastatin	No substantial variances in efficacy for each single loading vs. coloading in each nanoliposomes separately	Lack of potentiating of efficacies	8.3× fold enhancement	Newly emerging chemosensitisation of nanoliposomal atorvastatin pharmacologies
Atorvastatin–rutin co-loaded nanoliposomes vs. nanoliposomes of rutin	No substantial variances in efficacy for each single loading vs. coloading in each nanoliposomes separately	Lack of potentiating of efficacies	6.6× fold enhancement	Newly emerging potentiating of nanoliposomal Rutin pharmacologies
Atorvastatin–rutin co-loaded nanoliposomes vs. atorvastatin	5.3× fold augmentation of efficacy of atorvastatin	Effective chemosensitisation of atorvastatin potencies	55.4× fold increase in atorvastatin’s activity	Sustained chemosensitisation of atorvastatin affinities
Atorvastatin–rutin co-loaded nanoliposomes vs. rutin	No significant discrepancies in rutin’s efficacy	Lack of effective chemosensitisation of rutin potencies	25.78× fold increase in rutin’s activity	Newly emerging chemosensitisation of Rutin pharmacologies

**Table 5:** Targets, crystal structures and reference ligands.

Target	UniProt	PDB (Resolution, A)	Co-Crystallized Reference Ligand	Reference Binding Mode
BCL-2	P10415	6O0K (1.62)	Venetoclax	non-covalent
BCL-XL	Q07817	2YXJ (2.20)	ABT-737	non-covalent
MCL-1	Q07820	6FS1 (1.60)	Indole-acid MCL-1 inhibitor (AZD5991 series)	non-covalent
Caspase-3	P42574	2XYG (1.54)	CAS329306 (non-aspartate inhibitor)	covalent, Cys163
Caspase-7	P55210	1F1J (2.35)	Ac-DEVD-CHO	covalent, Cys186

**Table 6:** Head-to-head comparison. Redock RMSD is the pose retrieval of the reference ligand. The final three columns are Glide standard precision docking scores in kcal per mole, lower being better. Scores are comparable down a column within a target row, not across different targets.

Target	PDB (A)	Reference Ligand	Redock RMSD (A)	Rutin	Atorvastatin	Reference
BCL-2	6O0K (1.62)	Venetoclax	4.33	−5.09	−5.80	−9.32
BCL-XL	2YXJ (2.20)	ABT-737	13.70	−6.24	−5.74	−8.86
MCL-1	6FS1 (1.60)	AZD5991 series	1.20	−5.25	−6.51	−11.14
Caspase-3	2XYG (1.54)	CAS329306	3.23	−5.27	−5.34	−5.09
Caspase-7	1F1J (2.35)	Ac-DEVD-CHO	1.60	−5.59	−6.15	−10.76

**Table 7:** Contacts of the top-ranked pose of each test compound.

Target	Compound	Hydrogen Bonds	Hydrogen Bonds (Residue, Distance A)	Closest Hydrophobic Contacts
BCL-2	Rutin	5	ALA149 (2.35); ASP111 (2.12); GLU136 (1.9); GLU136 (1.83)	LEU137, GLU136, ALA149
BCL-2	Atorvastatin	1	ASN143 (2.13)	GLU136, PHE112, PHE104, ALA149, LEU137
BCL-XL	Rutin	4	ASP107 (1.77); ASP107 (1.9); GLU129 (2.01); ALA142 (2.11)	ALA104, GLU129, TYR101, PHE97, LEU130
BCL-XL	Atorvastatin	2	ARG139 (2.46); ARG139 (2.29)	LEU108, ALA104, PHE97, ARG139, GLY138
MCL-1	Rutin	4	THR266 (2.48); THR266 (2.75); ARG263 (2.67); ARG263 (2.72)	VAL249, VAL253, LEU235, THR266, PHE270
MCL-1	Atorvastatin	2	ARG263 (2.61); ARG263 (2.15)	PHE270, VAL249, MET250, PHE228, THR266
Caspase-3	Rutin	6	ASP253 (2.32); HIE121 (2.33); GLY122 (1.79); GLY122 (1.88)	PHE256, MET61, THR62, HIE121, TYR204
Caspase-3	Atorvastatin	3	HIE121 (1.92); CYS163 (2.16); ARG207 (2.72)	PHE128, MET61, THR166, HIE121, GLY122
Caspase-7	Rutin	8	ASP279 (2.0); SER231 (2.13); SER231 (1.72); GLN276 (1.98)	PHE282, PRO235, ARG233
Caspase-7	Atorvastatin	5	HIE144 (2.18); GLN184 (1.99); ARG87 (1.98); ARG233 (2.28)	HIE144, TRP232, PHE282, PRO235, GLY85

**Table 8:** Covalent docking of the two covalent reference ligands. These scores come from a different protocol from Table 6 and are not comparable with it.

Target	Covalent Reference Ligand	Attachment	Reaction	Affinity Score	Top-Pose RMSD (A)	Best-Pose RMSD (A)
Caspase-3	CAS329306	A:163 (Cys163)	Nucleophilic Substitution	−4.87	7.54	4.03
Caspase-7	Ac-DEVD-CHO	A:186 (Cys186)	Nucleophilic Addition to a Double Bond	−12.13	10.76	1.11

**Table 9:** Composition and formulation components of nanoliposomes.

Formulation Code	Lipid Composition & Molar Ratio HSPC: Cholesterol: PEG-lipid	Atorvastatin Content mg	Rutin Content mg	Solvent System 5 mL	Hydration Medium pH 7.4
Atorvastatin-loaded	65:30:5	2.0	—	Chloroform: Methanol 2:3 *v*/*v*	Phosphate-Buffered Saline
Rutin-loaded	65: 30:5	—	2.0	Chloroform: Methanol 2:3 *v*/*v*	Phosphate-Buffered Saline
Atorvastatin + RutinCo-loaded	65:30:5	2.0	2.0	Chloroform: Methanol 2:3 *v*/*v*	Phosphate-Buffered Saline

## Data Availability

The original contributions presented in this study are included in the article. Further inquiries can be directed to the corresponding author.

## Abbreviation

TCC: American Type Culture Collection, DLS: Dynamic Light Scattering, DPPH: 2,2-diphenyl-1-picrylhydrazyl (free radical assay), EE%: Encapsulation efficiency (percent), FEI: Field Electron and Ion, HPLC: High-performance liquid chromatography, HSPC: Hydrogenated soy L-α-phosphatidylcholine, IC_50_: Half-maximal inhibitory concentration, LPS: Lipopolysaccharide, SRB: Sulforhodamine B assay, PEG: Polyethylene glycol, PDI: Polydispersity index, PDL: Periodontal ligament fibroblasts, TEM: Transmission electron microscopy, UV: Ultraviolet.
